# Unicycler: Resolving bacterial genome assemblies from short and long sequencing reads

**DOI:** 10.1371/journal.pcbi.1005595

**Published:** 2017-06-08

**Authors:** Ryan R. Wick, Louise M. Judd, Claire L. Gorrie, Kathryn E. Holt

**Affiliations:** Department of Biochemistry and Molecular Biology, Bio21 Molecular Science and Biotechnology Institute, The University of Melbourne, Victoria, Australia; National Human Genome Research Institute, UNITED STATES

## Abstract

The Illumina DNA sequencing platform generates accurate but short reads, which can be used to produce accurate but fragmented genome assemblies. Pacific Biosciences and Oxford Nanopore Technologies DNA sequencing platforms generate long reads that can produce complete genome assemblies, but the sequencing is more expensive and error-prone. There is significant interest in combining data from these complementary sequencing technologies to generate more accurate “hybrid” assemblies. However, few tools exist that truly leverage the benefits of both types of data, namely the accuracy of short reads and the structural resolving power of long reads. Here we present Unicycler, a new tool for assembling bacterial genomes from a combination of short and long reads, which produces assemblies that are accurate, complete and cost-effective. Unicycler builds an initial assembly graph from short reads using the *de novo* assembler SPAdes and then simplifies the graph using information from short and long reads. Unicycler uses a novel semi-global aligner to align long reads to the assembly graph. Tests on both synthetic and real reads show Unicycler can assemble larger contigs with fewer misassemblies than other hybrid assemblers, even when long-read depth and accuracy are low. Unicycler is open source (GPLv3) and available at github.com/rrwick/Unicycler.

This is a *PLOS Computational Biology* Software paper.

## Introduction

Bacterial genomics is currently dominated by Illumina sequencing platforms. Illumina reads are accurate, have a low cost per base and have enabled widespread use of whole genome sequencing. However, much Illumina sequencing uses short fragments (500 bp or less) that are smaller than many repetitive elements in bacterial genomes[1]. This prevents short-read assembly tools (assemblers) from resolving the full genome, and their assemblies are instead fragmented into dozens of contiguous sequences (contigs). Consequently, most available bacterial genomes are incomplete, which hinders large-scale comparative genomic studies.

Pacific Biosciences (PacBio) and Oxford Nanopore Technologies (ONT) sequencing platforms can sequence DNA fragments of 10 kbp or longer, but at a higher cost per base than Illumina platforms. PacBio and ONT long reads also have much higher per-base error rates than Illumina reads (5–15% vs <1%), although they are often sufficient to complete bacterial genome assemblies with reasonable consensus accuracy[2,3]. Hence most researchers must choose between generating fragmented draft assemblies for many isolates with inexpensive Illumina sequencing, or generating complete assemblies for fewer isolates with expensive long-read technologies. Hybrid assembly, which uses a combination of short and long reads, offers an alternative. In this approach, short reads are used to produce accurate contigs and long reads provide the information to scaffold them together. This requires relatively few long reads and can thus be the most cost-effective route to a complete bacterial genome.

Despite recent developments in long-read technologies, Illumina reads are widely used in public health and research laboratories[4], and are likely to remain so for some time due to their high accuracy and low cost. Moreover, Illumina data is already available for hundreds of thousands of bacterial isolates, and most of these are unlikely to be replaced with long-read-only sequencing data. It is therefore probable that research and clinical labs will continue to use low cost Illumina reads for most samples and generate long reads as necessary to complete genomes of interest. Hybrid assembly, which requires fewer long reads than long-read-only assembly, is the most cost-effective means of achieving this goal.

Hybrid assembly can be accomplished with either a short-read-first or long-read-first approach. In the short-read-first method, a scaffolding tool uses long reads to join Illumina contigs together. However, scaffolding mistakes are common and lead to structural errors (misassemblies) in the sequence[5]. Long-read-first approaches may involve assembly of uncorrected long reads, followed by error-correction of the assembly using short reads[3]. Alternatively, they may first use short reads to correct errors in long reads, followed by assembly of the corrected long reads[6,7]. Whether error correction is performed before or after assembly, long-read-first approaches require higher long-read depth than short-read-first approaches.

Here we present Unicycler, a new hybrid assembly pipeline for bacterial isolate genomes. Unicycler first assembles short reads into an accurate and connected assembly graph, a data structure containing both contigs and their interconnections[8]. It then uses long reads to find the best paths through the graph. By following a short-read-first approach, Unicycler makes effective use of low quantities of long reads, but it can produce a completed assembly (one contig per replicon) if the long-read depth is sufficient. By using the assembly graph connections to constrain the possible scaffolding arrangements, Unicycler achieves lower misassembly rates than alternative short-read-first assemblers.

## Design and implementation

**Fig 1:**
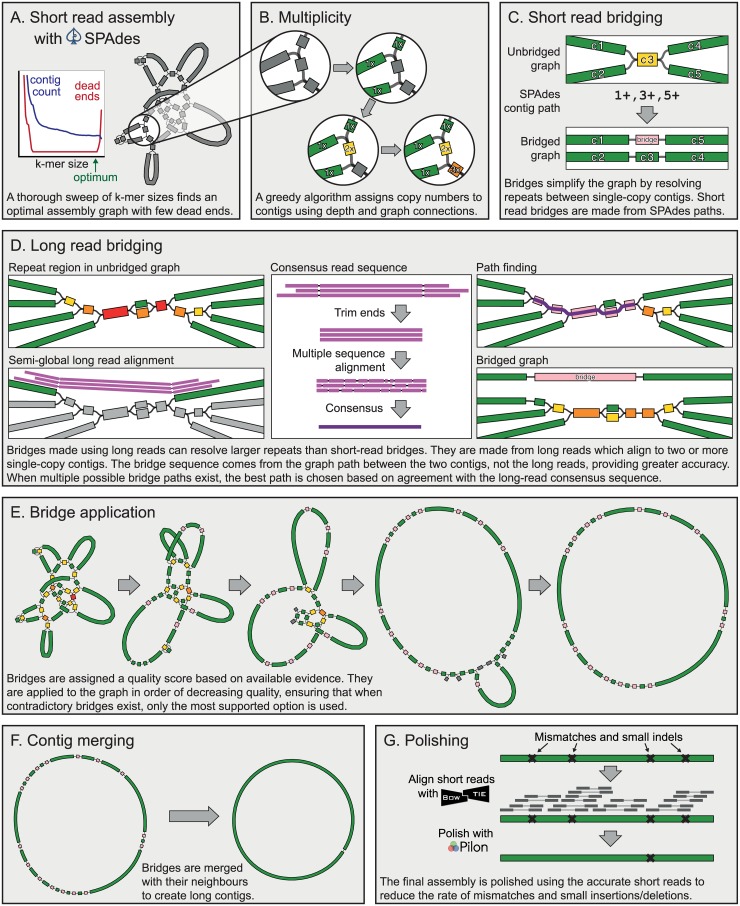
Key steps in the Unicycler pipeline.

Unicycler encapsulates its entire pipeline (Fig 1) in a single command and automatically determines low-level parameters so users can expect optimal results with default settings[9].

### Short-read assembly

Unicycler uses SPAdes (v3.6.2 or later) to construct a De Bruijn graph assembly using a wide range of k-mer sizes: 10 values spanning 20–95% of the Illumina read length (not exceeding 127, the largest k-mer possible in SPAdes)[10]. In SPAdes, large k-mers often result in larger contigs, but excessively large k-mers can cause a fragmented graph with dead ends. Unicycler assigns a score ($\frac{1}{c{\left(d+1\right)}^{2}}$) to each k-mer graph based on the number of contigs (*c*) and the number of dead ends (*d*). This score function penalises both large numbers of contigs and large numbers of dead ends. Since dead ends are particularly problematic in later Unicycler steps (see Multiplicity and Graph bridging using long-read alignments), the score function scales with the inverse of *d*^2^. The highest scoring graph is selected as a balance between minimising both contig count and dead ends (Figs 1A and S1).

As some contamination is possible in sequencing read sets (particularly when multiplexing, which is a common strategy for bacterial isolate sequencing on Illumina[11]), Unicycler then removes contigs with a depth of less than half the median graph depth, unless doing so would create a dead end. This removes most contamination while leaving important graph structures intact.

### Multiplicity

To resolve the graph as accurately as possible, Unicycler must first determine the multiplicity of contigs in the assembly graph. The most important distinction is between single-copy contigs (sequences that occur once in the genome, multiplicity *k* = 1) and repeat contigs (sequences that occur multiple times in the genome, multiplicity *k* > 1). However, determining the correct multiplicity for repeat contigs is also important, as this information can be used when finalising the assembly graph (see Conservative, normal and bold).

When a bacterial genome consists of a single chromosome with no additional replicons, then for each contig *x*, its median read depth *d*_x_ is a good indicator of its multiplicity *k*_x_. Single-copy contigs will have a median depth *d*_x_ close to *D*, the median depth per base across the entire assembly, while repeat contigs will have a median depth near a multiple of that value (i.e. *d*_x_ *~ k*_x_ *D*). The relationship between median read depth and multiplicity is more complicated when the genome contains multiple replicons present at different copy numbers per cell. For example, small plasmids are often present in multiple copies, while large conjugative plasmids are often present once per cell. The relationship between read depth and multiplicity only holds for replicons which exist in one copy per cell (the same as the chromosome). For example, contigs with depth *2D* may be chromosomal and have a multiplicity of two, or they may be in a two-copy-per-cell plasmid and have a multiplicity of one.

In addition to read depth, a contig’s graph connections also provide useful information about its multiplicity. Repeat contigs typically have multiple graph connections at their start and end, while single-copy contigs usually have only a single connection at each end. These trends break down when the assembly graph is fragmented, which is one reason why Unicycler aims to minimise the number of dead ends when determining the optimal short-read assembly graph.

To determine multiplicity values, Unicycler therefore uses both depth and connectivity information. Initially, a multiplicity of one is assigned to all contigs that are near the graph’s median depth and have no more than one connection at either end. A greedy algorithm then propagates multiplicity where graph connections and depth are in close agreement (Figs 1B and S2). When no more propagation is possible, the largest suitable contig is given a multiplicity of one and the process is repeated. This algorithm can correctly assign multiplicity to high-copy-number plasmid contigs in additional to chromosomal contigs.

### Bridges

Unicycler scaffolds assembly graphs by constructing bridge contigs to connect pairs of single-copy contigs. Before bridging, single-copy contigs connect via multiple alternative paths containing one or more repeat contigs. After bridging, they connect via a simple, unambiguous path. Bridges thus simplify the graph by resolving repeats. There are two primary sources of information available for creating bridges: paired-end short reads, which can resolve small repeats, and long reads, which can resolve much larger repeats.

### Graph bridging using read pair information

When SPAdes assembles paired-end reads, it uses its ExSPAnder algorithm to find paths through the assembly graph using read-pair orientation[12]. This process is known as repeat resolution (RR). SPAdes does not save its post-RR assembly (contigs.fasta) in graph form, but it does save the graph paths used to make post-RR contigs (contigs.paths). Unicycler finds cases where two single-copy contigs are connected in a SPAdes contig path and uses them to build bridges. In Fig 1C, the SPAdes contig path connects contigs 1 and 5 via contig 3. Unicycler’s resulting bridge connects contigs 1 and 5 directly with a copy of the contig 3 sequence. When this bridge is applied, contigs 2 and 4 also become connected via an unbranching path, in essence becoming bridged by process of elimination. These indirect graph simplifications may be merged together later in Unicycler’s pipeline, depending on the mode (see Conservative, normal and bold). While Unicycler creates bridges at this stage, they are not immediately applied to the graph. This is deferred to a later step where bridges are applied in decreasing order of quality (see Bridge application).

### Semi-global long-read alignment

While short reads can resolve repeats up to the insert size of the library (typically <1000 bp), long reads provide a much more powerful source of scaffolding information. As a first step in long-read bridging, Unicycler aligns all available long reads to the single-copy contigs. Since the long and short reads must be from the same biological sample, there should be no genuine structural discrepancies between the long reads and contigs. Semi-global alignment (i.e. end-gap free alignment) is therefore appropriate, where alignments can only terminate when the end of a sequence is reached. Most available long-read alignment tools such as BLASR[13], BWA-MEM[14], BLAST[15] and LAST[16] perform local alignment, so Unicycler implements semi-global alignment directly using the SeqAn C++ library (S3 Fig)[17].

### Graph bridging using long-read alignments

Long reads that align to multiple single-copy contigs can be used for bridging. Such reads contain a sample of the gap sequence between those contigs, and if multiple long reads connect a pair of contigs, Unicycler uses SeqAn to produce a consensus gap sequence[18,19]. Unicycler does not directly use this gap sequence in the bridge but instead uses it to find the best graph path connecting the contigs, via a branch and bound algorithm. Thus, the bridge sequence comes from the graph and reflects base calling accuracy of the short reads rather than the long reads that may have much lower accuracy (Fig 1D). Sometimes Unicycler cannot find a graph path connecting two single-copy contigs that are connected via long reads, such as when the short-read graph is incomplete and contains dead ends. In these cases, the long-read consensus sequence is directly used as the bridging sequence. Such bridges are more likely to contain errors—another reason why Unicycler strives to minimise dead ends in the assembly graph.

### Bridge application

Having produced bridges from both short reads (SPAdes RR) and long reads, Unicycler can now apply them to simplify the graph structure (Fig 1E). Since some bridges may be erroneous, Unicycler assigns a quality score to each bridge and applies them in order of decreasing quality, ensuring that when multiple contradictory bridges exist, the best-supported option is used.

Bridge quality is a function of many factors, depending on the type of bridge (S4 Fig). For long-read bridges, these factors are: the number of reads which support the bridge (more is better); the alignment quality between the read consensus and graph path (higher identity is better); the length of the two contigs to be bridged (longer is better); the length and quality of the read alignments to the contigs (longer and higher identity is better); and the read depth consistency between the contigs (closer agreement is better). An ideal long-read bridge therefore connects two long contigs of the same depth, is supported by many reads with long alignments to the contigs, and has a graph path in close agreement with the read sequences. See S4 Fig for more information on bridge quality functions.

### Final steps

Following bridge application, Unicycler performs several actions to finalise the assembly graph. Contigs that have been used in bridges and no longer provide additional connection information are removed. Simple unbranching paths in the graph are merged to form long contigs (Fig 1F). Overlapping sequences at contig ends (created by the SPAdes assembly process) are removed so each contig’s sequence leads directly into its neighbours. If any circular replicon was completely assembled, it will now be a single contig with a link connecting its end to its start. A circular sequence can be shifted to any starting position without changing the biological information. Unicycler therefore uses TBLASTN to search for *dnaA* or *repA* alleles in each completed replicon[20]. If one is found, the sequence is rotated and/or flipped so that it begins with that gene encoded on the forward strand. This provides consistently oriented assemblies and reduces the risk that a gene will be split across the start and end of the sequence. As a final step, Unicycler uses Bowtie2 and Pilon to polish the assembly using short-read alignments, reducing the rate of small errors (Fig 1G)[21,22].

### Conservative, normal and bold

Unicycler can be run in three different modes that alter its cutoff for minimum acceptable bridge quality: conservative, normal and bold. In conservative mode, the quality cutoff is high (i.e. only very high quality bridges will be used). This mode is least likely to produce a complete assembly but carries a very low risk of misassembly and is appropriate for contexts where assembly accuracy is paramount. In bold mode, the quality cutoff is low (i.e. lower quality bridges will be used). This mode is most likely to complete the assembly but carries greater risk of error. It is suited to cases where completeness is more important than accuracy. Normal mode uses an intermediate cutoff and is appropriate in most cases. The quality threshold value assigned to each mode (25, 10 and 1 for conservative, normal and bold, respectively) is arbitrary and the user can manually specify a different threshold for finer control. Furthermore, in conservative mode, Unicycler excludes all SPAdes contig bridges, as tests reveal SPAdes RR to be a common source of misassembly (see Assembly of simulated short-read datasets).

Unicycler’s mode also influences its contig-merging behaviour in bridge finalisation. In bold mode, all possible contigs on unbranching paths are merged, regardless of their multiplicity and whether they are bridges. In conservative mode, Unicycler only merges single-copy contigs and their corresponding bridges. Simple paths indirectly created by bridging (as is the case with contigs 2 and 4 in Fig 1C) will not be merged in conservative mode. In normal mode, single-copy contigs can also be merged with non-bridge contigs, but only if their multiplicity is one. For example, contig 3 in Fig 1C has a multiplicity of two before bridge application and a single instance has been used in the bridge, leaving the contig with a multiplicity of one after bridge application. Unicycler would therefore merge this path (contigs 2, 3 and 4) in normal mode.

### Included tools

Unicycler’s semi-global alignment algorithm is included as a stand-alone command line tool (unicycler_align), making it available for use in other pipelines. Unicycler also comes with a polishing tool (unicycler_polish) which applies variants identified by Pilon, GenomicConsensus[2] and FreeBayes[23], and assesses the assembly using ALE[24]. By iteratively polishing the genome with both short and long reads, this process can correct many remaining errors in a completed assembly, including those in repeat regions.

## Results

Unicycler’s performance was evaluated using read sets simulated for eight species and using real read sets from the well-studied *E*. *coli* K-12 *substr*. MG1655. We further demonstrated the utility of Unicycler by assembling the complete genomes of novel isolates of *Klebsiella pneumoniae* using newly generated Illumina, PacBio and ONT reads.

ABySS does not perform hybrid assembly and was only used in the short-read-only tests. npScarf and Cerulean require long reads and were only used for the hybrid-read tests. SPAdes can assemble with or without long reads and was included in all tests. We used default parameters or recommended settings for all tools (S1 Table). The NaS tool can conduct hybrid assemblies but was excluded from this comparison because it depends on Newbler, a closed-source assembler only supported on RedHat/Fedora Linux[25]. We also excluded ALLPATHS-LG, which can perform hybrid assemblies but has strict library preparation requirements, restricting its applicability[26,27].

### Metrics

For both the simulated and real *E*. *coli* read tests, assemblies were evaluated by comparison to the corresponding complete reference genome using QUAST (v4.3)[28]. We focused on the following metrics: misassemblies, small-error rate (mismatches and small indels) and NGA50.

QUAST identifies misassemblies as cases where a contig aligns to the reference genome in multiple pieces, not as a single continuous alignment, indicating a structural error in the contig. QUAST distinguishes between “local” and “extensive” misassemblies: local misassemblies have a discrepancy of less than 1 kbp while extensive misassemblies have a larger discrepancy. For our tests, we used the sum of both types to quantify all misassemblies, regardless of size. For the simulated read tests, reads were generated from the reference genome so misassemblies always indicate assembler mistakes. For the *E*. *coli* tests, there is not a perfect agreement between the reference genome and reads generated in different laboratories from different subcultures of *E*. *coli* K-12 *substr*. MG1655, so false positive misassemblies are possible.

The well-known N50 metric measures only contig size, not contig correctness, limiting its value. A large N50 can therefore result from inaccurately joining sequences into large misassembled contigs. By aligning contigs to a reference, QUAST produces more useful metrics including NGA50 (GA = “genome aligned”). Whereas N50 is based on contig lengths, NGA50 is based on the lengths of contig-to-reference alignments. A correctly assembled contig will have a single, unbroken alignment to the reference; a misassembled contig will be divided into multiple smaller alignments. Hence NGA50 is a metric for completeness that, unlike N50, penalises misassemblies. In our tests, we used QUAST’s “strict-NA” option to break contigs at all misassembly locations, including local misassemblies, for particularly stringent NGA50 scores.

### Simulated read sets

To provide a wide range of genome size and complexity, we simulated reads from 12 reference genomes from seven bacterial species (2 *Acinetobacter baumannii*[29,30], 2 *Escherichia coli*[31,32], 3 *Klebsiella pneumoniae*[33–35], 1 *Mycobacterium tuberculosis*[36], 1 *Shigella dysenteriae*[37], 1 *Shigella sonnei*, 1 *Streptococcus suis*[38]) and the yeast *Saccharomyces cerevisiae*[39] (Table 1). Plasmid and mitochondrial sequences were included at higher read depths, as appropriate.

**Table 1:** Reference genomes for simulated read sets.

Species	Strain	Genome size (bp)	GC content	Description	MLST sequence type	Other features	GenBank assembly accession
*Acinetobacter baumannii*	A1	3,917,739	39.3%	Circular chromosome, one plasmid	231	Large, repetitive biofilm-associated protein gene	GCA_000830055.1
*Acinetobacter baumannii*	AB30	4,335,793	39.0%	Circular chromosome	758	Large, repetitive biofilm-associated protein gene	GCA_000746645.1
*Escherichia coli*	K-12 MG1655	4,641,652	50.8%	Circular chromosome	10		GCA_000005845.2
*Escherichia coli*	O25b:H4-ST131 EC958	5,249,449	50.8%	Circular chromosome, two plasmids	131		GCA_000285655.3
*Klebsiella pneumoniae*	30660/NJST258_1	5,540,936	57.2%	Circular chromosome, five plasmids	258		GCA_000598005.1
*Klebsiella pneumoniae*	MGH 78578	5,694,894	57.1%	Circular chromosome, five plasmids	38		GCA_000016305.1
*Klebsiella pneumoniae*	NTUH-K2044	5,472,672	57.4%	Circular chromosome, one plasmid	23		GCA_000009885.1
*Mycobacterium tuberculosis*	H37Rv	4,411,532	65.6%	Circular chromosome		High-copy-number PE and PPE genes	GCA_000195955.2
*Saccharomyces cerevisiae*	S288c	12,157,105	38.1%	16 linear chromosomes, circular mitochondrial sequence		Eukaryote	GCA_000146045.2
*Shigella dysenteriae*	Sd197	4,560,911	51.0%	Circular chromosome, two plasmids	146	High insertion sequence content	GCA_000012005.1
*Shigella sonnei*	53G	5,220,473	50.7%	Circular chromosome, four plasmids	152	High insertion sequence content	GCA_000283715.1
*Streptococcus suis*	BM407	2,170,808	41.0%	Circular chromosome, one plasmid	1		GCA_000026745.1

We used ART (v2.5.8) to generate six synthetic paired-end short-read sets from each reference genome which mimic those from an Illumina HiSeq 2500: 125 bp read length, 400 bp mean insert size, 60 bp insert size standard deviation and 50x read depth[40,41]. Each synthetic short-read set was assigned a long-read accuracy and mean length: 60% and 10 kbp; 60% and 25 kbp; 75% and 10 kbp; 75% and 25 kbp; 90% and 10 kbp; and 90% and 25 kbp. For each short-read set, we used PBSIM[42] to generate synthetic long reads at seven depths (0.25x, 0.5x, 1.0x, 2.0x, 4.0x, 8.0x and 16.0x). This yielded six short-read sets and 42 hybrid-read sets per strain. For the short-read sets, we performed five assemblies: Unicycler in each of its modes (conservatives, normal and bold), SPAdes and ABySS. For the hybrid sets, we performed six assemblies: Unicycler (all modes), SPAdes, npScarf and Cerulean. Additionally, all tests were performed in five replicates using separately generated synthetic reads, resulting in 16920 total assemblies.

### Assembly of simulated short-read datasets

**Fig 2:**
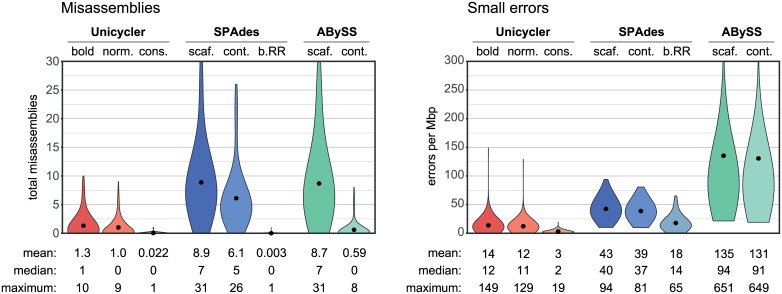
Simulated short-read assemblies: Errors. Misassembly and small-error (mismatches and indels) rates for assemblies of simulated short-read sets, summarising results across all reference genomes and replicate tests (total 360 per assembler).

**Fig 3:**
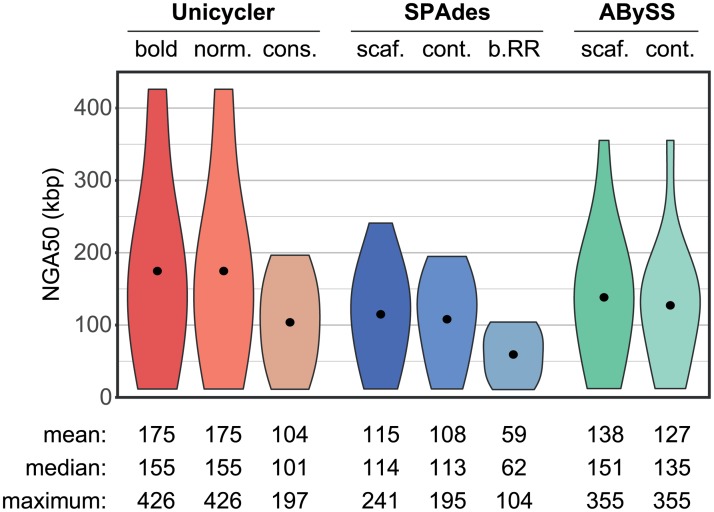
Simulated short-read assemblies: NGA50. NGA50 for assemblies of simulated short-read sets, summarising results across all reference genomes and replicate tests (total 360 per assembler).

For assemblies of synthetic short-read-only sets, Unicycler outperformed the other assemblers in each QUAST metric (Figs 2 and 3, S2 Table). It is particularly interesting to compare Unicycler to SPAdes, since Unicycler uses SPAdes to build the initial short-read assembly graph.

In normal and bold modes, Unicycler achieved the most complete assemblies, as measured by NGA50. This is attributable to the wide k-mer range used in assembly. Both SPAdes and ABySS allow for manual selection of k-mer size, and providing a higher k-mer would likely improve the contiguity of their assemblies. However, we tested each assembler using the settings recommended in the tool’s documentation or provided in example commands. SPAdes was run without defined k-mer sizes, and for the test read sets it automatically selected k21–55. ABySS was run with a k-mer of 64 (the maximum value with default compilation settings). Unicycler’s automatically-selected k-mer differed between read sets, but was most typically 95, giving it greater power to assemble repetitive regions than SPAdes and ABySS.

The misassembly rate of pre-RR SPAdes assemblies was very low, demonstrating that RR is the source of most misassemblies in SPAdes contigs. In conservative mode, Unicycler does not use SPAdes RR and therefore achieves similarly low misassembly rates. In normal and bold modes, Unicycler does use RR, but only if it exceeds a quality threshold. This selectiveness explains why normal/bold Unicycler assemblies have lower misassembly rates than the SPAdes contig assemblies from which they are derived.

Both Unicycler and SPAdes incorporate a polishing step into their pipeline. Unicycler uses Bowtie2 and Pilon. SPAdes uses MismatchCorrector, which is optional but we included it in our tests because the SPAdes documentation recommends it for small genomes. This polishing likely accounts for Unicycler’s and SPAdes’ superior performance in the small-errors metric. ABySS assemblies may show similarly low small-error rates if a subsequent polishing step was performed.

### Hybrid assembly of simulated short- and long-read datasets

**Fig 4:**
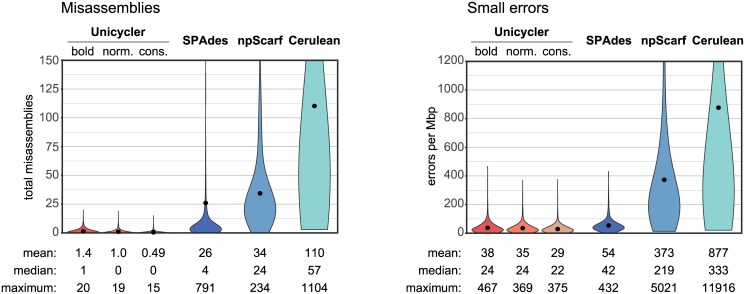
Simulated hybrid assemblies: Errors. Error rates for hybrid assemblies of simulated short-read and long-read sets, summarising results across all reference genomes and replicate tests (total 2520 per assembler).

Unicycler surpassed other assemblers when conducting hybrid assemblies of synthetic reads (S2 Table). Misassembly rates in the hybrid assemblies were often much higher than in the short-read-only assemblies, illustrating the difficulty of resolving repeats with long reads (Figs 4, S5 and S6). Both npScarf and Cerulean consistently produced assemblies with ten or more misassemblies. SPAdes produced fewer misassemblies, but some genomes resulted in many errors, particularly the *Shigella* genomes with many high-copy-number ~1 kbp repeats associated with insertion sequences. Unicycler’s misassembly rates were the lowest and correlated with the assembly mode (conservative, normal or bold).

**Fig 5:**
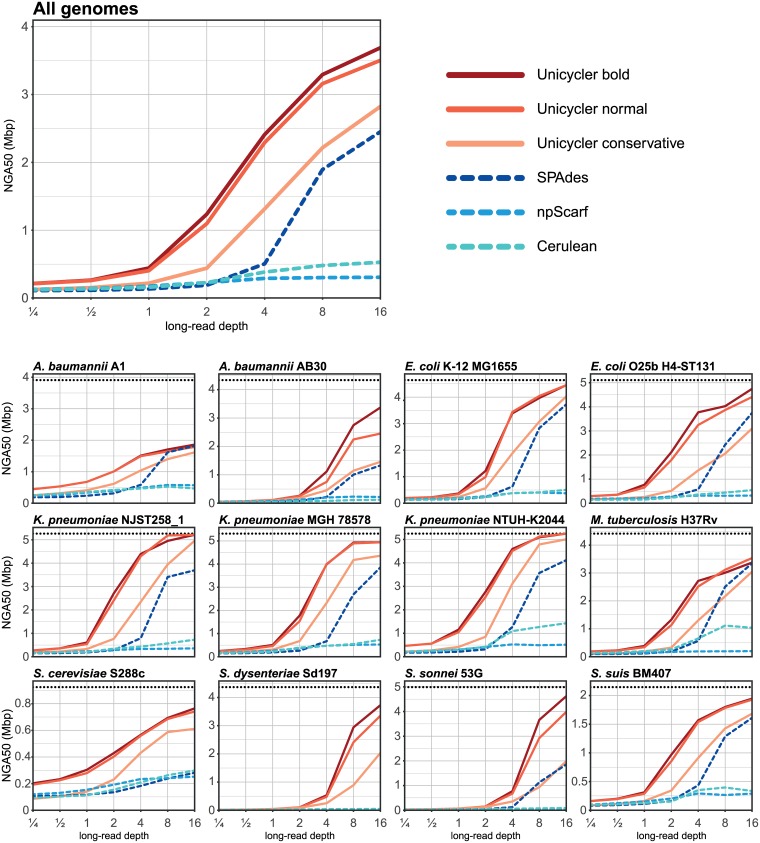
Simulated hybrid assemblies: NGA50 against long-read depth. Mean NGA50 values for hybrid assemblies of simulated read sets. Mean values were calculated across all read lengths, read accuracies and replicate tests for each reference genome (210 hybrid-read sets each); the top panel shows mean values for all 12 reference genomes (2520 hybrid-read sets). Horizontal dashed lines indicate the N50 size of the reference genome. For the bacterial genomes, this is the size of their only chromosome; for *Saccharomyces*, it is the size of chromosome XIII, an intermediate-sized replicon in the genome.

Small-error rates (mismatches and small indels) were lowest in Unicycler and SPAdes, as they both derive their final contigs from the short-read assembly graph, not from the long-read sequences. Unicycler’s and SPAdes’ polishing steps may also contribute to their low small-error rate. NGA50 was dependent on the long-read depth, and Unicycler performed best at all tested depths (Fig 5). This is due to Unicycler’s low misassembly rates (other assemblers’ NGA50 scores were reduced due to their higher occurrence of misassemblies) and its ability to produce bridges using as few as one long read. In many cases, Unicycler produced complete or near-complete assemblies with only 4x long-read depth.

**Fig 6:**
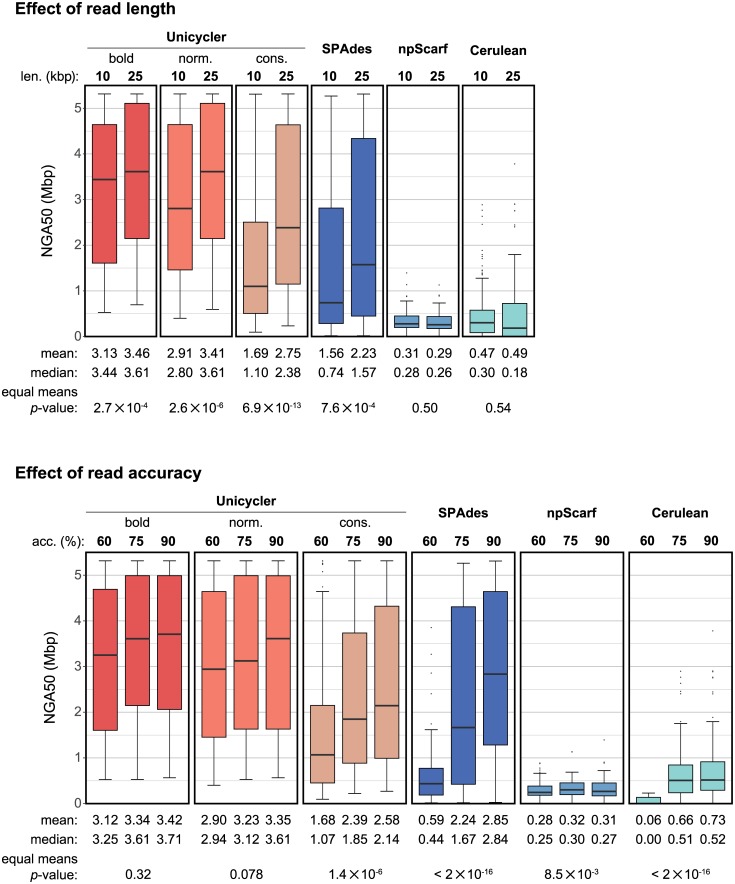
Simulated hybrid assemblies: Read length and accuracy. NGA50 values segregated by read length and read accuracy. These plots summarise results across all reference genomes and replicate tests, but only include the tests of 8x long-read depth. For read lengths, the *p*-value is from a two-tailed *t*-test. For read accuracies, the *p*-value is from a one-way ANOVA test.

Theoretical analyses of assembly show that error-prone reads are nearly as informative as error-free reads, suggesting that read accuracy is less important than length[43,44]. Unicycler’s performance on the simulated read sets matched these findings. Read length significantly affected the resulting NGA50 for Unicycler (all modes) and SPAdes (Fig 6). In contrast, read accuracy had a weaker effect on Unicycler’s NGA50 values, demonstrating its effectiveness in using long reads regardless of their accuracy.

### Computational performance

The assembly tests were all conducted with eight CPU cores and 16 GB of RAM. Unicycler was slower than the alternative hybrid assemblers, taking a median time of 46 minutes to assemble the 8x long-read depth synthetic tests. SPAdes and npScarf performed the fastest, both having a median time of eight minutes and maximum time of less than 25 minutes on the same data. Cerulean had a median time of 23 minutes, although some Cerulean processes did not complete due to crashes or exceeding the 24-hour time limit. The complex biofilm-associated gene in *Acinetobacter baumannii* A1 was slow to bridge in Unicycler, resulting in a maximum run time of 13 hours. However, Unicycler did fully assemble this gene sequence in many read sets where the other assemblers produced a fragmented or misassembled result.

### Real *E*. *coli* K-12 read sets

We tested the same assemblers using real reads of the *E*. *coli* K-12 *substr*. MG1655 genome. The short reads for these tests were produced on the Illumina MiSeq platform. Long reads were from five different platforms: ONT R7, ONT R9, PacBio RS, PacBio RS II C2 chemistry and PacBio RS II C3 chemistry (Table 2). The ONT R9 reads were further split into two groups, pass and fail, as determined by ONT’s Metrichor software (v0.16.37960). For each platform and long-read depth, we conducted 20 trials using different random subsamples of long reads at the same depths used for simulated data. Accuracy was assessed by comparison to the *E*. *coli* K-12 *substr*. MG1655 reference genome (accession NC_000913.3) generated using Sanger-based capillary sequencing at the University of Wisconsin in 1997[31].

**Table 2:** Real *E*. *coli* K-12 *substr*. MG1655 read sets.

Platform	Read count	Total length (Mbp)	Aligned length (Mbp)	Mean depth	Median length (bp)	N50 length (bp)	Mean identity (%)	Availability
Illumina MiSeq	2,200,000	584.7	581.7	125.3	300	300	99.4	www.illumina.com/systems/miseq/scientific_data.html
ONT R7	68,455	311.5	151.4	32.6	3,751	7,290	67.7	www.ncbi.nlm.nih.gov/sra/?term=ERR637419
ONT R9 (fail)	24,649	148.2	118.7	25.6	6,729	9,230	75.8	lab.loman.net/2016/07/30/nanopore-r9-data-release/
ONT R9 (pass)	50,277	328.8	280.7	60.5	7,669	9,315	81.0	lab.loman.net/2016/07/30/nanopore-r9-data-release/
PacBio RS, C2 enzyme C2 chemistry	42,582	106.8	105.1	22.6	2,016	4,030	85.0	github.com/PacificBiosciences/DevNet/wiki/E-coli-K12-MG1655-Resequencing
PacBio RS II, P4 polymerase C2 chemistry	82,590	437.0	432.7	93.2	4,589	7,566	80.7	www.ncbi.nlm.nih.gov/sra/SRX669475
PacBio RS II, P5 polymerase C3 chemistry	47,910	396.8	395.7	85.3	7,613	11,789	85.0	www.ncbi.nlm.nih.gov/sra/SRX533603

**Fig 7:**
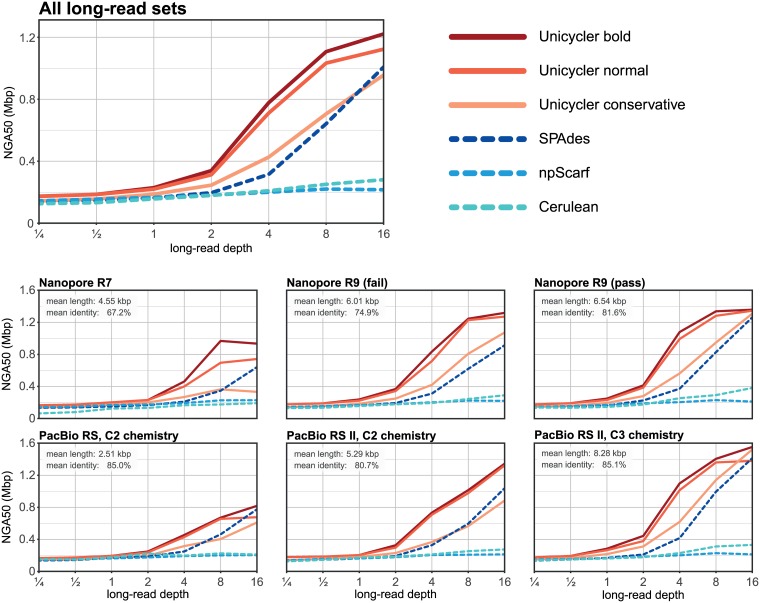
*E*. *coli* K-12 assemblies: NGA50 against long-read depth. Mean NGA50 values for hybrid assemblies of real *E*. *coli* read sets, summarised across 20 replicate tests at each depth. Top panel shows mean values for all six long-read sets.

These tests showed the same trends as the simulated data: Unicycler produced larger contigs at lower long-read depths than other assemblers (Fig 7, S3 Table). Notably, Unicycler performed worst on the set with shortest reads (PacBio RS) not the set with lowest read identity (ONT R7), while it performed best on the set with longest reads (PacBio RS II C3). This matches the simulated results and theoretical predictions, again showing that read length is more important than accuracy for Unicycler.

We also searched for each of the *E*. *coli* K-12 *substr*. MG1655 reference genome’s seven RNA operons in the assemblies using BLAST. These repetitive sequences are approximately 5 kbp in length and have enough sequence similarity to each other to create fragmented short-read assemblies[45]. We produced seven query sequences from the reference genome: each RNA operon along with 2 kbp of neighbouring sequence on each end. If an operon had a BLAST hit exceeding both 95% identity and 95% coverage, then we counted it as found in the assembly. Each assembly could therefore achieve a score from zero (no complete RNA operons found) to seven (all RNA operons found). Unicycler performed best in this analysis, often finding all seven operons when it used 8x or 16x depth of long reads (S8 Fig).

The NGA50s for these tests were markedly lower than those obtained with reads simulated from the *E*. *coli* K-12 *substr*. MG1655 reference genome. With simulated reads, both Unicycler and SPAdes were often able to achieve complete or near-complete assemblies. With real reads, Unicycler’s and SPAdes’ best NGA50 values were 2.0 Mbp and 1.4 Mbp, respectively. This appears to be due to false positive misassembly calls resulting from genuine differences between the reference genome sequence and the genomes of the isolates that were sequenced using Illumina, PacBio and ONT platforms (S7 Fig). For example, one copy of IS*1*A in the Illumina read set relocated to a position 105 kbp away from the position in the reference genome; when an assembly spanned this region, QUAST identified the difference as a misassembly, reducing the NGA50.

### *Klebsiella pneumoniae* PacBio assembly case study

To explore the utility of Unicycler, we used it to assemble the genome of *K*. *pneumoniae* isolate INF274, which was difficult to assemble using alternative techniques. INF274 is a multidrug-resistant sequence type (ST) 340 strain isolated from the urine of a Melbourne hospital patient who had a urinary tract infection. It belongs to clonal group (CG) 258, a common cause of multidrug-resistant hospital-associated infections globally. This isolate was first sequenced on Illumina HiSeq 2000 (generating 750 Mbp of 125 bp paired-end reads) and then on a PacBio RS II (generating 1.3 Gbp of long reads, many of which exceeded 10 kbp in length). Both reads sets are high quality and are an ideal set of inputs for hybrid assembly. Notably, the library preparation for the PacBio reads followed a standard size-selection protocol that excluded short fragments of DNA, so small plasmids were underrepresented in the long reads.

This sample’s PacBio read set was sufficiently deep for both hybrid and long-read-only assembly approaches. For hybrid assembly, we used Unicycler (normal mode) and SPAdes (v3.8.1), the best performing assemblers in our synthetic tests. For long-read-only assemblies, we used HGAP (v3) and Canu (v1.3), both of which are modern implementations of the Celera Assembler designed for high-error long reads[2,3,46]. HGAP was included because it is developed by Pacific Biosciences and included in their SMRT Analysis software suite. Canu is a similar assembler designed for both PacBio and ONT reads.

**Fig 8:**
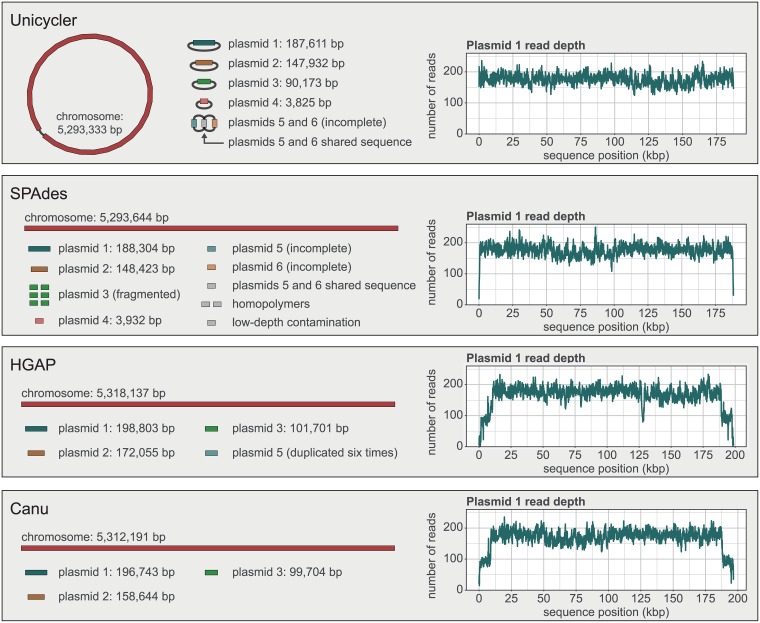
*Klebsiella pneumoniae* INF274 assembler comparison. Final assemblies of *Klebsiella pneumoniae* INF274 produced by Unicycler, SPAdes, HGAP and Canu. The contigs/graph of the assembly are shown on the left, coloured by replicon. The read depth plot of plasmid 1’s contig is shown on the right. Low read depth at the ends of the contig is indicative of start-end overlap.

Since INF274 is a novel isolate, we did not analyse the assembly results with QUAST. Instead, we qualitatively compared the assemblies and analysed the alignment of Illumina reads to each (Figs 8 and S9). Of the four assemblers, only Unicycler and Canu produce a graph file for their final assembly, but Canu did not circularise any replicons, so the sequences remained linear. When viewed in Bandage[8], the Unicycler graph clearly distinguished between replicons that formed completed circularised sequences and those that did not. Only plasmids 5 and 6 remained incomplete in the Unicycler assembly, as they contain shared sequence and there was an absence of long reads for these replicons, preventing Unicycler from scaffolding them apart. SPAdes and HGAP output their assemblies only as linear sequences, making it difficult to make the distinction between complete and incomplete replicons. The SPAdes assembly suffered from the same problem as Unicycler with plasmids 5 and 6, and it failed to assemble plasmid 3, which contains a prophage sequence also present in the chromosome.

Since Unicycler’s graph-based scaffolding naturally results in circular sequences, it did not have duplicated sequences at the start/end of circular replicons. SPAdes suffered from a slight overlap and both HGAP and Canu had significant overlaps, indicated by the drop in read depth near the ends of contigs. These must be repaired manually or with a tool such as Circlator[47].

### *Klebsiella pneumoniae* ONT assembly case study

To assess Unicycler’s performance on low-depth ONT datasets, we performed R9 sequencing on *K*. *pneumoniae* isolate INF125, a virulent ST45 strain isolated from the urine of a Melbourne hospital patient. Reads were generated over a four-hour period resulting in a total of 156 Mbp of sequence (depth = 28.6x) with an N50 length of 11,470 bp. ONT streams sequence data as it is generated, making it feasible to analyse the data in real time and stop sequencing when a complete assembly is reached.

To investigate each assembler’s suitability for such real-time analysis, we generated 240 subsets of reads, one set per minute of sequencing, each containing all reads generated up to that minute (e.g. set 60 contained all reads generated in the first hour of sequencing). All sets were assembled with Unicycler (normal mode), SPAdes, npScarf and miniasm. Unicycler and SPAdes were included due to their high accuracy in the synthetic read tests. npScarf was included because it is explicitly designed for streaming, real-time analysis. Miniasm is a long-read-only assembler which, unlike HGAP and Canu, does not produce a consensus sequence[48]. Rather, its assemblies consist of read fragments, have an error rate similar to the raw reads, and require consensus improvement using a separate tool, such as Racon[49]. It was included in these tests because of its speed—it only takes a few minutes to run—making it potentially suitable for real-time analysis.

**Fig 9:**
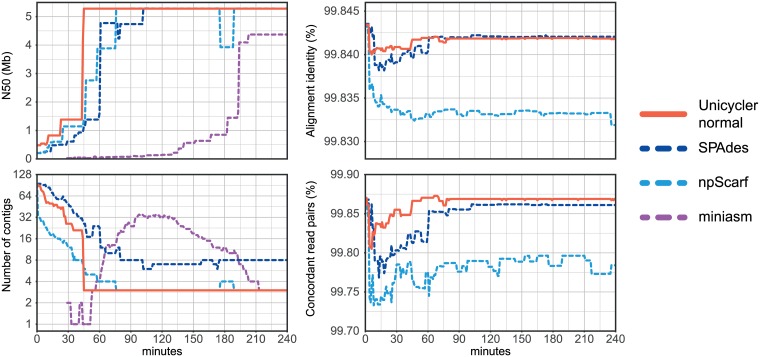
*Klebsiella pneumoniae* INF125 ONT assemblies over sequencing time. Assembly metrics of *K*. *pneumoniae* INF125 produced by Unicycler, SPAdes, npScarf and miniasm over a four-hour period of sequencing. Miniasm assemblies contain error rates comparable to that of the raw reads and are therefore excluded from the error rate plots.

We assessed each assembly using N50, number of contigs, and error rates when aligning the Illumina reads to the assembly (Fig 9, S4 Table). A high read alignment identity is indicative of a low small-error rate (mismatches and small indels). A high proportion of concordantly aligned reads is indicative of a low misassembly rate. Due to the intrinsically high error rates of its uncorrected assemblies, miniasm was excluded from the read alignment tests.

Unicycler completed the INF125 assembly at with 45 minutes of ONT reads (depth = 5.3x). npScarf required 76 minutes of reads (9.0x) to complete the assembly, SPAdes took 102 minutes of reads (12.1x) and miniasm took 213 minutes of reads (25.3x). All completed assemblies contained three contigs (one chromosome and two plasmids), except for SPAdes assemblies which contained extra, erroneous contigs. Read error metrics show that both Unicycler and SPAdes consistently produced more accurate assemblies than npScarf, although the magnitude of the benefit was small (Fig 9).

### Limitations

As Unicycler operates on a short-read assembly graph, it requires high quality short reads. Specifically, it is important that there are very few unsequenced regions of the genome that create dead ends in the assembly graph. The quality of Unicycler’s assemblies can suffer if the assembly graph is fragmented and incomplete.

### Summary

Unicycler performed well on both short-read-only sets and all types of hybrid-read sets, producing larger contigs than other assemblers. Perhaps more importantly, Unicycler produced fewer misassemblies than other assemblers, which often had high error rates. As long-read sequencing becomes more common, so will completed genome assemblies, enabling new research into genome structure. High quality assemblies free of structural errors, such as those produced by Unicycler, will be critical to research in this field.

## Availability and future directions

Unicycler’s primary use case is when a researcher wishes to complete the assembly of an isolate for which Illumina reads already exist. To facilitate this, future development of Unicycler will add streaming support for ONT sequencing, using reads to create and update bridges in the graph in real time during a sequencing run. This will allow users to halt sequencing once a genome is sufficiently resolved, conserving sequencing resources for other isolates. This modality is currently possible with npScarf, however in our tests Unicycler was more accurate than npScarf and reached complete assemblies with lower read depths. Future development will also focus on improving Unicycler’s computational performance. Unicycler is not currently able to perform large assemblies such as human genomes and metagenomes. Algorithmic improvements to long-read alignment, path finding and graph manipulations will all be required for Unicycler to be appropriate in such cases.

Unicycler is open source (GPLv3) and available at github.com/rrwick/Unicycler.

## Supporting information

S1 Fig Effect of maximum SPAdes k-mer on short-read assemblies. As the maximum assembly k-mer grows, SPAdes assemblies have decreasing numbers of segments (top row). The assembly graphs have fewest dead ends for moderate k-mers (middle row). Unicycler chooses its ideal maximum k-mer using a score function which takes both segments and dead ends into account (bottom row).
(PDF)

S2 Fig Unicycler’s multiplicity algorithm and an example of its application on a simple assembly graph. (PDF)

S3 Fig Unicycler’s semi-global alignment algorithm and an example of its application on a read-contig pair. (PDF)

S4 Fig Bridge quality score functions. All bridges in Unicycler are assigned a quality score, which allows Unicycler to sort bridges by quality. Quality scores are calculated as the product of multiple score functions, and then transformed into the range 0 to 100. Each score function quantifies some aspect of the bridge in the range of 0 and 1, and different bridge types use different combinations of these functions in their quality score. The score functions are heuristic, not statistically derived.
(PDF)

S5 Fig Simulated hybrid assemblies: Misassemblies per genome. Misassembly rates for hybrid assemblies of simulated short-read and long-read sets, summarising results separately for each reference genome (total 210 results per assembler per reference).
(PDF)

S6 Fig Simulated hybrid assemblies: Small errors per genome. Small-error rates for hybrid assemblies of simulated short-read and long-read sets, summarising results separately for each reference genome (total 210 results per assembler per reference).
(PDF)

S7 Fig *E*. *coli* K-12 assemblies: Errors. Error rates for hybrid assemblies of real *E*. *coli* read sets, summarised across 840 results per assembler.
(PDF)

S8 Fig *E*. *coli* K-12 assemblies: RNA operons against long-read depth. Number of RNA operons found in hybrid assemblies of real *E*. *coli* read sets, summarised across 840 results per assembler.
(PDF)

S9 Fig *Klebsiella pneumoniae* INF274 assembly errors. Error rates when aligning Illumina reads to each *K*. *pneumoniae* INF274 assembly. An increase in discordant pairs is indicative of a misassembly. An increase in error rate is indicative of small errors (mismatches and small indels).
(PDF)

S10 Fig *Klebsiella pneumoniae* INF125 Illumina assembly comparison. Comparison of assembly graphs made by different assemblers using only short reads for *K*. *pneumoniae* INF125. Contigs are coloured based on which replicon they represent.
(PDF)

S1 Table Commands used for assembly, evaluation and read simulation. (XLSX)

S2 Table Raw QUAST results for simulated read tests. (TSV)

S3 Table Raw QUAST results for *E*. *coli* read tests. (TSV)

S4 Table Raw quality metrics for *K*. *pneumoniae* INF125 ONT assemblies over time. (TSV)
