# Supplementary figures and images for: Unicycler: Resolving bacterial genome assemblies from short and long sequencing reads
Source: PLoS Comput Biol. 2017 Jun 8;13(6):e1005595. doi: 10.1371/journal.pcbi.1005595 (PMC5481147; doi:10.1371/journal.pcbi.1005595)

*Klebsiella pneumoniae* INF125

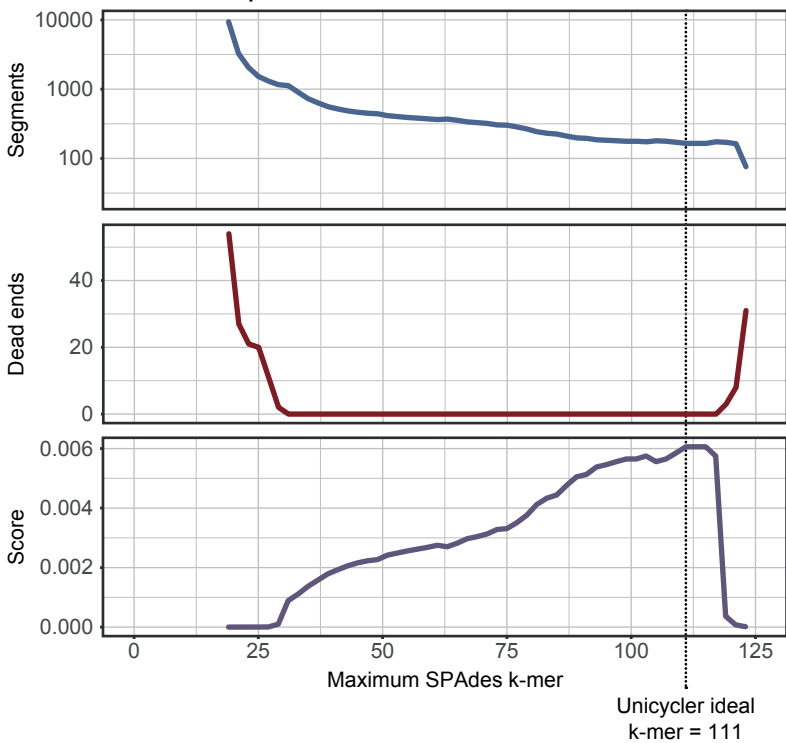

*Klebsiella pneumoniae* INF274

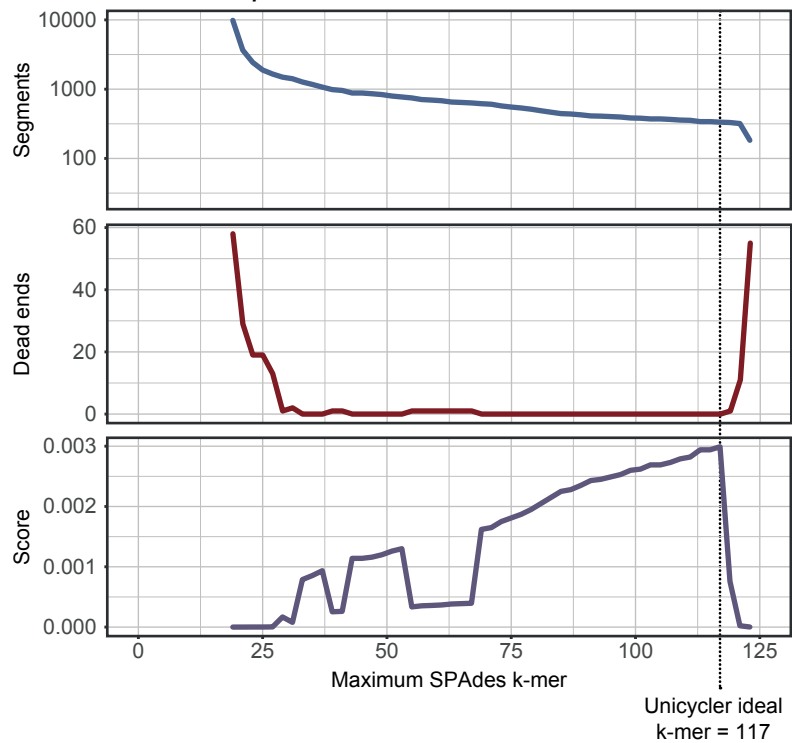

Supplement: S1 Fig — As the maximum assembly k-mer grows, SPAdes assemblies have decreasing numbers of segments (top row). The assembly graphs have fewest dead ends for moderate k-mers (middle row). Unicycler chooses its ideal maximum k-mer using a score function which takes both segments and dead ends into account (bottom row). (PDF) [file pcbi.1005595.s001.pdf]

# Semi-global read alignment in Unicycler-align

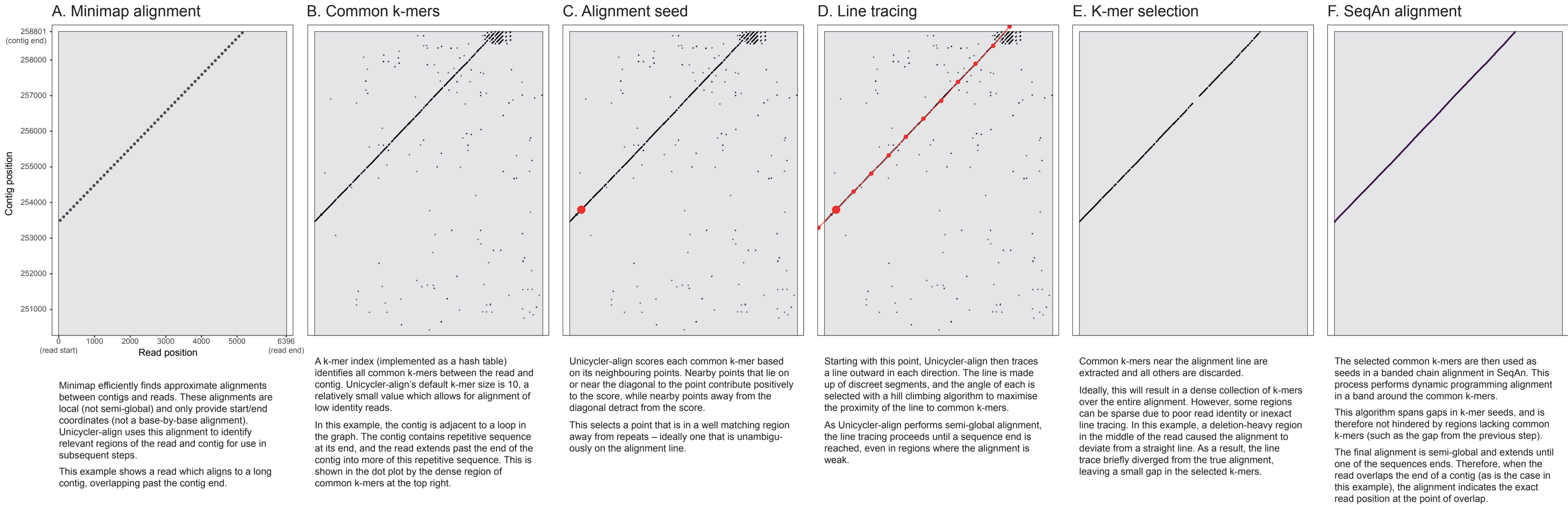

## Comparison of long-read alignment tools

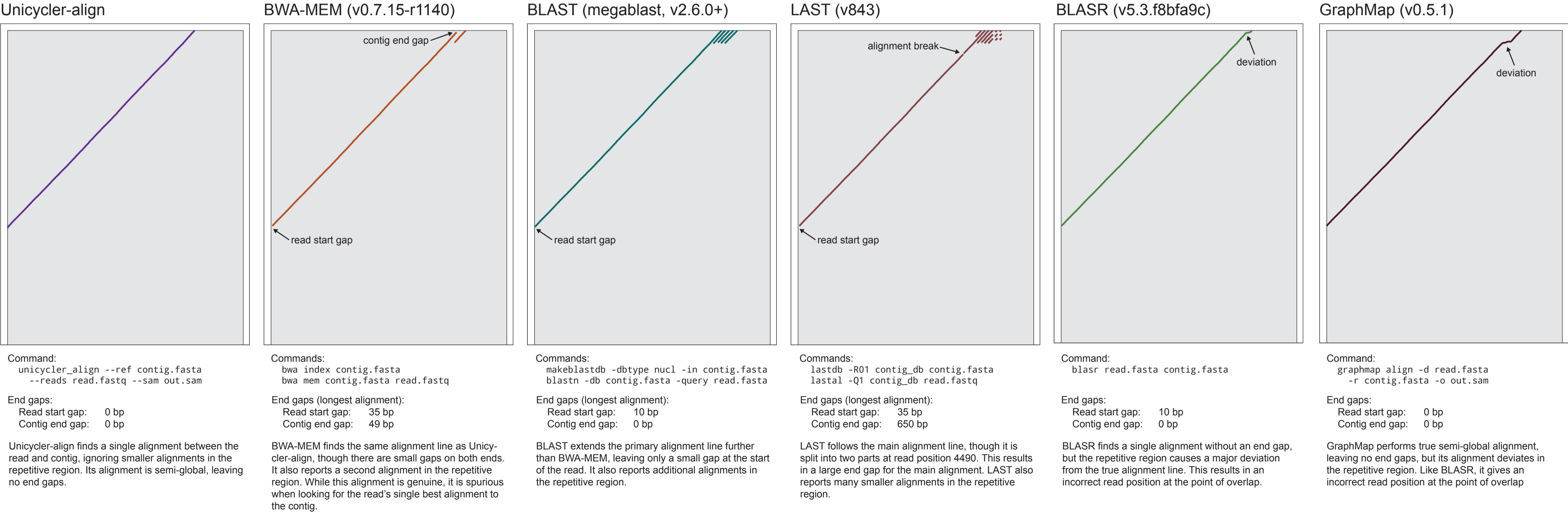

Supplement: S3 Fig — (PDF) [file pcbi.1005595.s003.pdf]

# Misassemblies

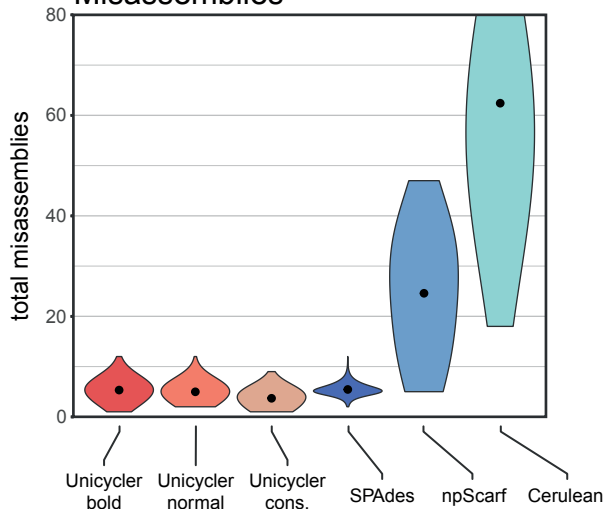

|          |     |     |     |     |    |     |
|----------|-----|-----|-----|-----|----|-----|
| mean:    | 5.3 | 5.0 | 3.7 | 5.4 | 25 | 62  |
| median:  | 5   | 5   | 4   | 5   | 26 | 58  |
| maximum: | 12  | 12  | 9   | 12  | 47 | 241 |

# Small errors

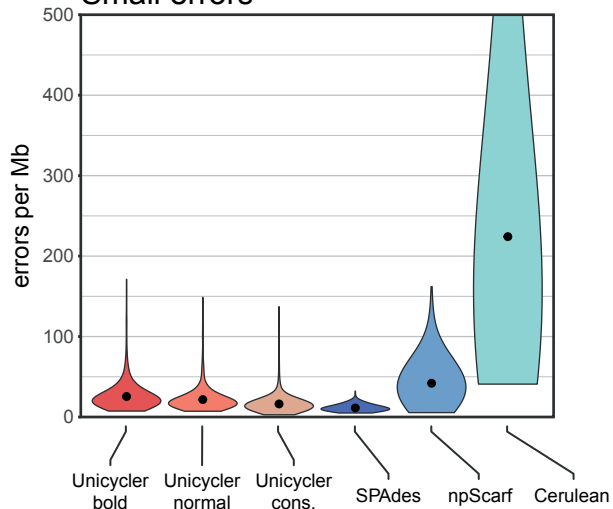

|          |     |     |     |    |     |     |
|----------|-----|-----|-----|----|-----|-----|
| mean:    | 26  | 22  | 16  | 11 | 42  | 224 |
| median:  | 21  | 18  | 14  | 10 | 40  | 175 |
| maximum: | 171 | 149 | 137 | 32 | 162 | 947 |

Supplement: S7 Fig — Error rates for hybrid assemblies of real E. coli read sets, summarised across 840 results per assembler. (PDF) [file pcbi.1005595.s007.pdf]

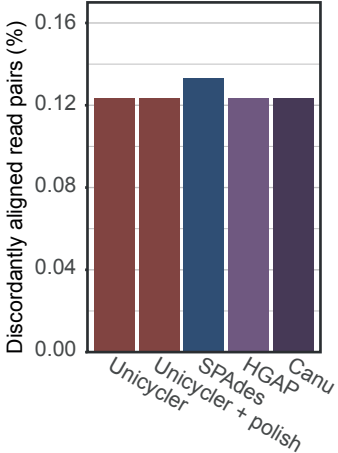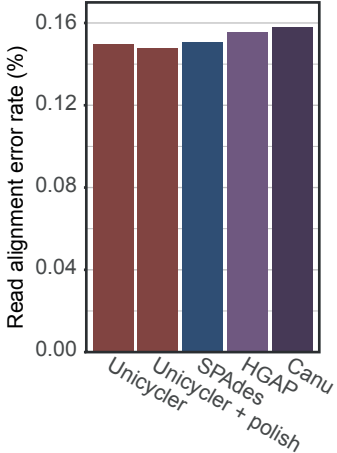

Supplement: S9 Fig — Error rates when aligning Illumina reads to each K. pneumoniae INF274 assembly. An increase in discordant pairs is indicative of a misassembly. An increase in error rate is indicative of small errors (mismatches and small indels). (PDF) [file pcbi.1005595.s009.pdf]
