# Supplementary material for: Unicycler: Resolving bacterial genome assemblies from short and long sequencing reads
Source: PLoS Comput Biol. 2017 Jun 8;13(6):e1005595. doi: 10.1371/journal.pcbi.1005595 (PMC5481147; doi:10.1371/journal.pcbi.1005595)
Supplement: S4 Fig — All bridges in Unicycler are assigned a quality score, which allows Unicycler to sort bridges by quality. Quality scores are calculated as the product of multiple score functions, and then transformed into the range 0 to 100. Each score function quantifies some aspect of the bridge in the range of 0 and 1, and different bridge types use different combinations of these functions in their quality score. The score functions are heuristic, not statistically derived. (PDF) [file pcbi.1005595.s004.pdf]

Bridge types

SPAdes contig bridge

Unicycler makes SPAdes contig bridges from paths in SPAdes' contigs.paths file. These bridges connect two single-copy contigs with the contigs in the path.

$$\text{score} = 100\sqrt{0.4dcl}$$

Example

single-copy

SPAdes contig path: 13+,2-,24+,13+,5+,18-

Bridge: 2→24→13→5

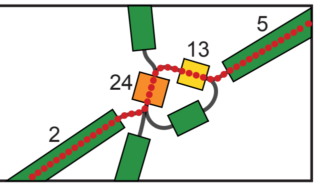

Loop unrolling bridge

Loop unrolling bridges are a special case of SPAdes contig bridge for when a SPAdes contig path connects a single-copy contig to the middle contig of a loop. In such cases, Unicycler concludes that the loop is contiguous with the contig and uses the contig depths to determine the number of times to traverse the loop.

$$\text{score} = 100\sqrt{0.4dwq}$$

Example

SPAdes contig path: 1+,14+,18-

Mean single-copy depth: $\frac{1.0+1.1}{2} = 1.05$

Loop count by contig 14: $\frac{2.9-1}{1.05} = 1.81$

Loop count by contig 18: $\frac{2.2}{1.05} = 2.10$

Final loop count: $\frac{1.81+2.10}{2} = 1.95$

Bridge: 1→14→18→14→18→14→3

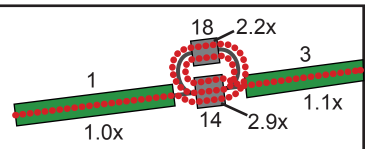

Long-read bridge with path

Unicycler finds long reads which connect two single-copy contigs and uses them to form a long-read bridge. It then searches for a graph path corresponding to the long-read consensus sequence. If a graph path is found, that sequence is used for the bridge instead of the long-read consensus sequence.

$$\text{score} = 100\sqrt{drgmns}$$

Example

Bridge: 1→41→25→21→31→36→7

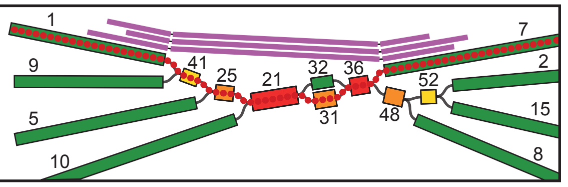

Long-read bridge without path

When Unicycler cannot find a graph path corresponding to a bridge's long-read consensus sequence (either due to poor homology or the absence of a path), it uses the consensus sequence directly.

This approach is less desirable, as the long-read consensus is likely to contain more errors than the short-read graph. However, it is necessary in cases when the short-read graph is incomplete and contains dead ends.

$$\text{score} = 100\sqrt{drgmne}$$

Example

Bridge: 1→read consensus→7

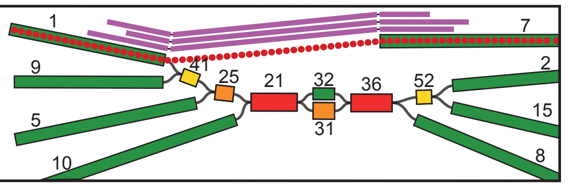

Score functions

Depth agreement: d

applies to all bridge types

$$d(x,y) = \frac{1}{1+10^{2\left(\log\left(\frac{\max\{x,y\}}{\min\{x,y\}}\right)-1\right)+0.45}}$$

x = contig 1 depth y = contig 2 depth

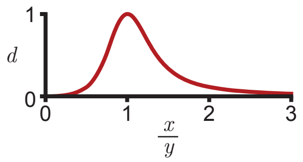

Good case

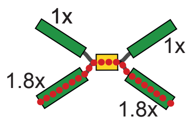

Single-copy contigs have the same depth (1.8x).

Bad case

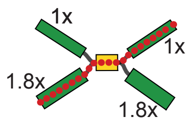

Single-copy contigs have differing depths (1x vs 1.8x).

The *d* function quantifies how well the two contigs being bridged agree in read depth. A close agreement is good, as it suggests the contigs are from the same replicon. A poor agreement is bad, as it may indicate the contigs are from different replicons.

Depth consistency: c

applies to SPAdes contig bridges

$$c(u,v,x,y,\text{path}) = \begin{cases} \prod_{k \text{ in path}} a(k_{\text{dep}}, z(u,v,x,y)k_{\text{count}}) & \text{if path is self-contained,} \\ 1 & \text{otherwise} \end{cases}$$

$$z(u,v,x,y) = x\frac{u}{u+v} + y\frac{v}{u+v} \qquad a(i,j) = \frac{\min\{i,j\}}{\max\{i,j\}}$$

u = contig 1 length v = contig 2 length x = contig 1 depth y = contig 2 depth

k_{dep} = contig in path depth k_{count} = occurrences of contig in path

Good case

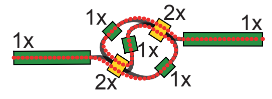

Contig depths and path counts are consistent (e.g. 2x depth contigs are traversed twice).

Bad case

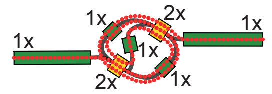

Contig depths and path counts are inconsistent (e.g. 2x depth contigs are traversed three times).

The *c* function quantifies the consistency of depth and count for contigs in the path. It only applies to self-contained paths, i.e. paths where all contigs lead exclusively to the single-copy contigs being bridged. In such cases, there should be a close agreement between contig depth and count, and this function penalises cases where the agreement is poor.

Bridge length: l

applies to SPAdes contig bridges

$$l(h,p_\mu,p_\sigma) = \begin{cases} \frac{p_\sigma}{h-p_\mu+p_\sigma} & \text{if } h > p_\mu, \\ 1 & \text{otherwise} \end{cases}$$

h = bridge length p_μ = paired-end mean insert size

p_σ = paired-end insert size standard deviation

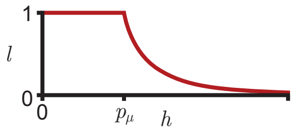

Good case

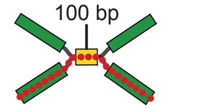

Bridge is shorter than the typical insert size.

Bad case

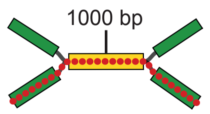

Bridge is longer than the typical insert size.

SPAdes contig bridges which span repeats longer than the typical insert size are not reliable, as it is unlikely paired-end reads could resolve such a repeat. This function penalises excessively long SPAdes contig bridges.

Whole loop number: w

applies to loop unrolling bridges

$$w(o) = \begin{cases} o & \text{if } o < 1, \\ 1-2(0.5-|0.5-o+\lfloor o \rfloor|) & \text{otherwise} \end{cases}$$

o = loop count (not rounded)

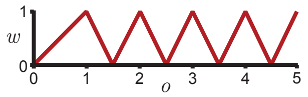

This function penalises loop counts that are between two integers, as such cases may be the higher count (rounding up) or lower count (rounding down).

Good case

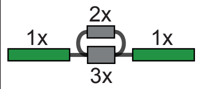

Loop count is an integer.

$o = 2$

Bad case

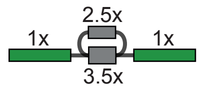

Loop count is between integers.

$o = 2.5$

Loop count penalty: q

applies to loop unrolling bridges

$$q(o_{\text{rounded}}) = \frac{1}{2^{o_{\text{rounded}}-1}}$$

o_{rounded} = loop count (rounded to nearest integer)

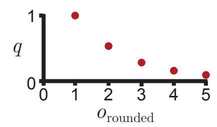

Good case

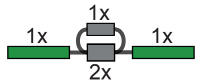

Low loop count, easy to call.

Bad case

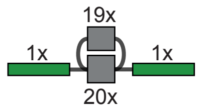

High loop count, hard to call.

Low loop counts are easier to distinguish due to a larger relative difference in depth (e.g. 1x vs 2x). High loop counts have a smaller relative difference in depth (e.g. 19x vs 20x) and are therefore harder to distinguish. This function penalises bridges with larger loop counts, as their exact count is less certain.

Read count: r

applies to all long-read bridges

$$r(n_{\text{actual}}, n_{\text{expected}}) = \min\left\{1, \frac{n_{\text{actual}}}{n_{\text{expected}}}\right\}$$

n_{actual} = actual number of long reads in bridge

n_{expected} = expected number of long reads in bridge

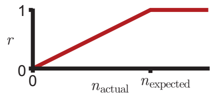

The *r* function penalises bridges with fewer than expected reads. The expected value assumes an even read distribution over the entire genome size.

Good case

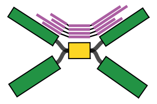

Expected number of reads support the bridge.

Bad case

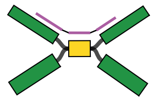

Fewer than expected number of reads support the bridge.

Contig alignment: m

applies to all long-read bridges

$$m(i_{\text{start}}, i_{\text{end}}) = \frac{\min\left\{\max\{i_{\text{start}}\}, \max\{i_{\text{end}}\}\right\}}{100}$$

i_{start} = set of alignment identities to contig 1

i_{end} = set of alignment identities to contig 2

Good case

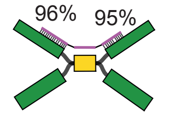

High-identity alignments to bridged contigs.

Bad case

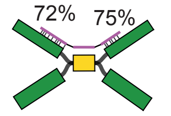

Low-identity alignments to bridged contigs.

Misalignments of long reads (i.e. long reads aligning to non-homologous contigs) are less likely with high identity alignments. The *m* function rewards bridges with high-identity read alignments and punishes bridges with low-identity read alignments.

Path alignment: s

applies to long-read bridges with path

$$s(c_{\text{actual}}, c_{\text{expected}}) = \frac{1}{1+2^{c_{\text{expected}}-c_{\text{actual}}}}$$

c_{actual} = actual identity between long read consensus and bridge path

c_{expected} = expected identity between long read consensus and bridge path

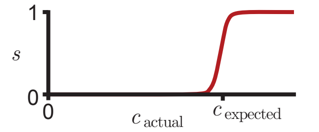

Good case

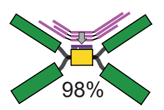

High identity between consensus and bridge path.

Bad case

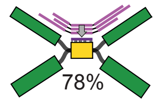

Low identity between consensus and bridge path.

If a bridge's read consensus aligns poorly to the graph path, this suggests the graph path may not be homologous with the reads. The *s* function penalises bridges where the read consensus has a low alignment identity to the graph path.

Alignment length: g

applies to all long-read bridges

$$g(a_{\text{start}}, a_{\text{end}}, m) = 1 - \frac{4m}{4m + \min\left\{\max\{a_{\text{start}}\}, \max\{a_{\text{end}}\}\right\}}$$

a_{start} = set of alignment lengths to contig 1

a_{end} = set of alignment lengths to contig 2

m = minimum alignment length

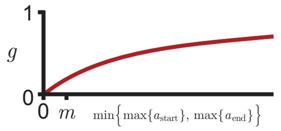

Good case

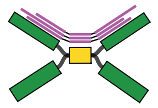

Long alignments to bridged contigs.

Bad case

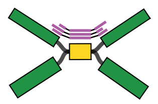

Short alignments to bridged contigs.

Misalignments of long reads (i.e. long reads aligning to non-homologous contigs) are less likely with longer alignments. The *g* function rewards bridges with longer alignments and punishes bridges with shorter alignments.

Contig length: n

applies to all long-read bridges

$$n(l_{\text{start}}, l_{\text{end}}, m) = \min\left\{\frac{4m}{4m+l_{\text{start}}}, \frac{4m}{4m+l_{\text{end}}}\right\}$$

l_{start} = length of contig 1

l_{end} = length of contig 2

m = minimum alignment length

Good case

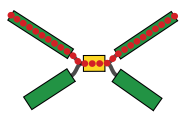

Bridge connecting long contigs.

Bad case

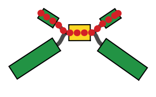

Bridge connecting short contigs.

Short contigs are more likely to be falsely identified as single-copy (i.e. actually have a multiplicity > 1). The *n* function therefore rewards bridges between long contigs, as these are more likely to be bridges between genuinely single-copy contigs.

Dead ends: e

applies to long-read bridges without path

$$e(d) = \begin{cases} 1 & \text{if } d = 2, \\ 0.7 & \text{if } d = 1, \\ 0.2 & \text{otherwise} \end{cases}$$

d = total number of dead ends at the end of contig 1 and the start of contig 2 (0, 1 or 2)

Good case

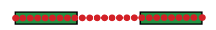

Pathless bridge connects two dead-end contigs.

Bad case

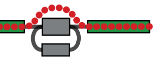

Pathless bridge connects contigs without dead-ends.

The *e* function penalises long-read bridges without paths if the bridge does not span contig dead ends. This discourages the use of a pathless long-read bridge when a true path exists.
