# Supplementary material for: Unicycler: Resolving bacterial genome assemblies from short and long sequencing reads
Source: PLoS Comput Biol. 2017 Jun 8;13(6):e1005595. doi: 10.1371/journal.pcbi.1005595 (PMC5481147; doi:10.1371/journal.pcbi.1005595)
Supplement: S5 Fig — Misassembly rates for hybrid assemblies of simulated short-read and long-read sets, summarising results separately for each reference genome (total 210 results per assembler per reference). (PDF) [file pcbi.1005595.s005.pdf]

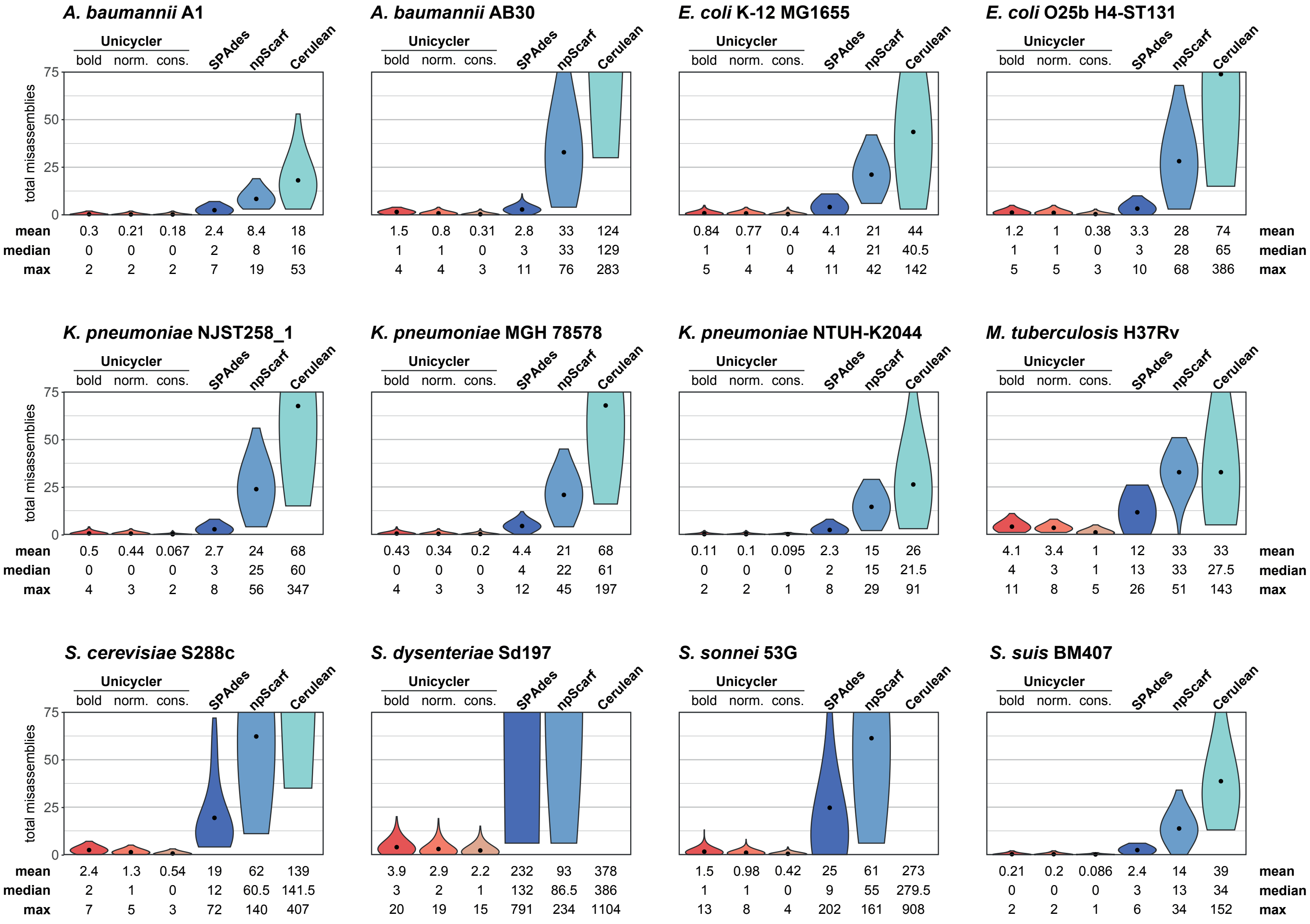

A. baumannii A1
Unicycler
bold norm. cons.
SPAdes npScarf Cerulean
75 50 25 0
total misassemblies
mean 0.3 0.21 0.18 2.4 8.4 18
median 0 0 0 2 8 16
max 2 2 2 7 19 53
A. baumannii AB30
Unicycler
bold norm. cons.
SPAdes npScarf Cerulean
mean 1.5 0.8 0.31 2.8 33 124
median 1 1 0 3 33 129
max 4 4 3 11 76 283
E. coli K-12 MG1655
Unicycler
bold norm. cons.
SPAdes npScarf Cerulean
mean 0.84 0.77 0.4 4.1 21 44
median 1 1 0 4 21 40.5
max 5 4 4 11 42 142
E. coli O25b H4-ST131
Unicycler
bold norm. cons.
SPAdes npScarf Cerulean
mean 1.2 1 0.38 3.3 28 74
median 1 1 0 3 28 65
max 5 5 3 10 68 386
K. pneumoniae NJST258_1
Unicycler
bold norm. cons.
SPAdes npScarf Cerulean
75 50 25 0
total misassemblies
mean 0.5 0.44 0.067 2.7 24 68
median 0 0 0 3 25 60
max 4 3 2 8 56 347
K. pneumoniae MGH 78578
Unicycler
bold norm. cons.
SPAdes npScarf Cerulean
mean 0.43 0.34 0.2 4.4 21 68
median 0 0 0 4 22 61
max 4 3 3 12 45 197
K. pneumoniae NTUH-K2044
Unicycler
bold norm. cons.
SPAdes npScarf Cerulean
mean 0.11 0.1 0.095 2.3 15 26
median 0 0 0 2 15 21.5
max 2 2 1 8 29 91
M. tuberculosis H37Rv
Unicycler
bold norm. cons.
SPAdes npScarf Cerulean
mean 4.1 3.4 1 12 33 33
median 4 3 1 13 33 27.5
max 11 8 5 26 51 143
S. cerevisiae S288c
Unicycler
bold norm. cons.
SPAdes npScarf Cerulean
75 50 25 0
total misassemblies
mean 2.4 1.3 0.54 19 62 139
median 2 1 0 12 60.5 141.5
max 7 5 3 72 140 407
S. dysenteriae Sd197
Unicycler
bold norm. cons.
SPAdes npScarf Cerulean
mean 3.9 2.9 2.2 232 93 378
median 3 2 1 132 86.5 386
max 20 19 15 791 234 1104
S. sonnei 53G
Unicycler
bold norm. cons.
SPAdes npScarf Cerulean
mean 1.5 0.98 0.42 25 61 273
median 1 1 0 9 55 279.5
max 13 8 4 202 161 908
S. suis BM407
Unicycler
bold norm. cons.
SPAdes npScarf Cerulean
mean 0.21 0.2 0.086 2.4 14 39
median 0 0 0 3 13 34
max 2 2 1 6 34 152
