# Supplementary material for: Unicycler: Resolving bacterial genome assemblies from short and long sequencing reads
Source: PLoS Comput Biol. 2017 Jun 8;13(6):e1005595. doi: 10.1371/journal.pcbi.1005595 (PMC5481147; doi:10.1371/journal.pcbi.1005595)
Supplement: S6 Fig — Small-error rates for hybrid assemblies of simulated short-read and long-read sets, summarising results separately for each reference genome (total 210 results per assembler per reference). (PDF) [file pcbi.1005595.s006.pdf]

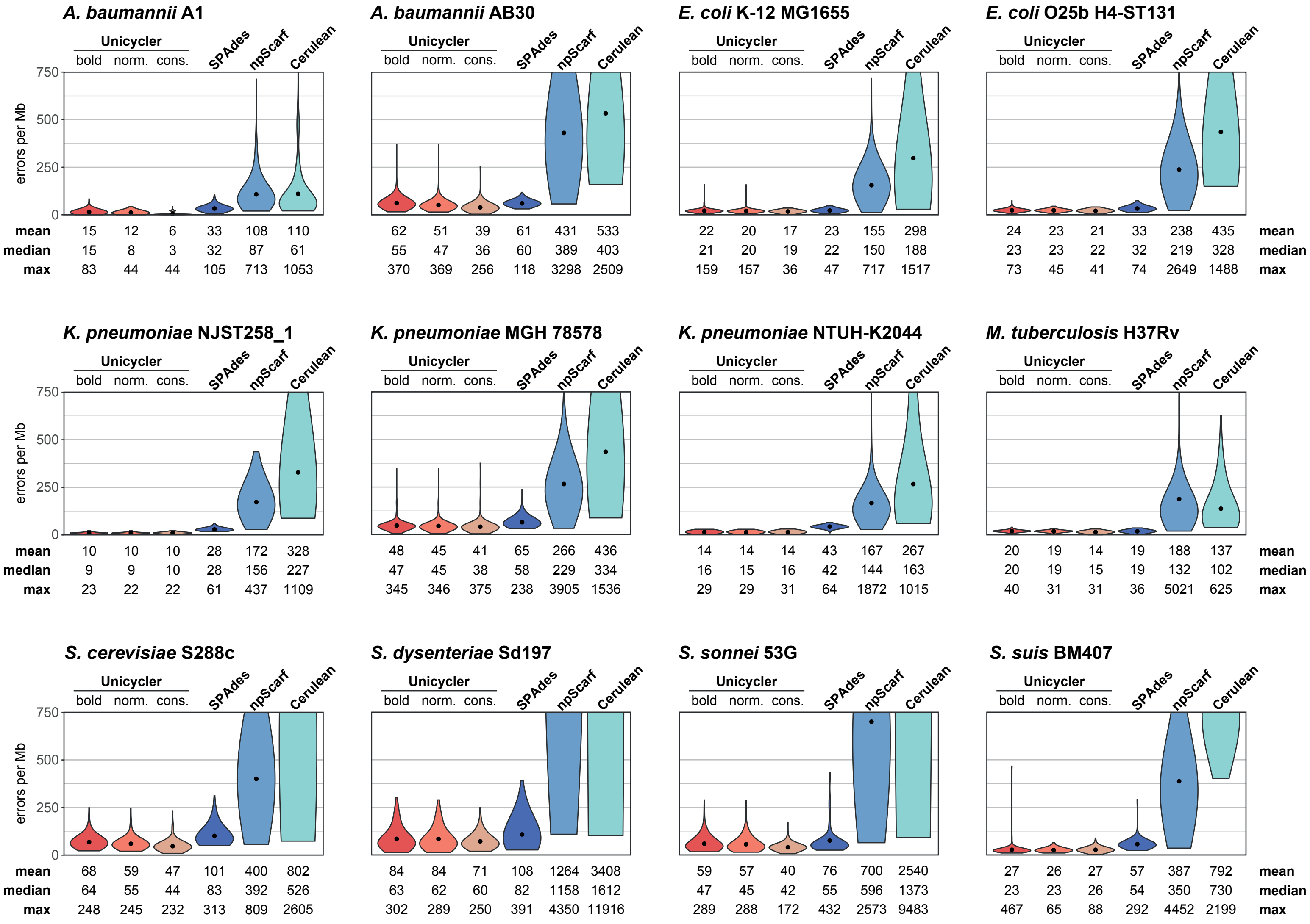
A. baumannii A1
Unicycler
bold norm. cons.
SPAdes npScarf Cerulean
errors per Mb
750 500 250 0
mean 15 12 6 33 108 110
median 15 8 3 32 87 61
max 83 44 44 105 713 1053
A. baumannii AB30
mean 62 51 39 61 431 533
median 55 47 36 60 389 403
max 370 369 256 118 3298 2509
E. coli K-12 MG1655
mean 22 20 17 23 155 298
median 21 20 19 22 150 188
max 159 157 36 47 717 1517
E. coli O25b H4-ST131
mean 24 23 21 33 238 435
median 23 23 22 32 219 328
max 73 45 41 74 2649 1488
K. pneumoniae NJST258_1
mean 10 10 10 28 172 328
median 9 9 10 28 156 227
max 23 22 22 61 437 1109
K. pneumoniae MGH 78578
mean 48 45 41 65 266 436
median 47 45 38 58 229 334
max 345 346 375 238 3905 1536
K. pneumoniae NTUH-K2044
mean 14 14 14 43 167 267
median 16 15 16 42 144 163
max 29 29 31 64 1872 1015
M. tuberculosis H37Rv
mean 20 19 14 19 188 137
median 20 19 15 19 132 102
max 40 31 31 36 5021 625
S. cerevisiae S288c
mean 68 59 47 101 400 802
median 64 55 44 83 392 526
max 248 245 232 313 809 2605
S. dysenteriae Sd197
mean 84 84 71 108 1264 3408
median 63 62 60 82 1158 1612
max 302 289 250 391 4350 11916
S. sonnei 53G
mean 59 57 40 76 700 2540
median 47 45 42 55 596 1373
max 289 288 172 432 2573 9483
S. suis BM407
mean 27 26 27 57 387 792
median 23 23 26 54 350 730
max 467 65 88 292 4452 2199
