# Supplementary material for: Unicycler: Resolving bacterial genome assemblies from short and long sequencing reads
Source: PLoS Comput Biol. 2017 Jun 8;13(6):e1005595. doi: 10.1371/journal.pcbi.1005595 (PMC5481147; doi:10.1371/journal.pcbi.1005595)
Supplement: S8 Fig — Number of RNA operons found in hybrid assemblies of real E. coli read sets, summarised across 840 results per assembler. (PDF) [file pcbi.1005595.s008.pdf]

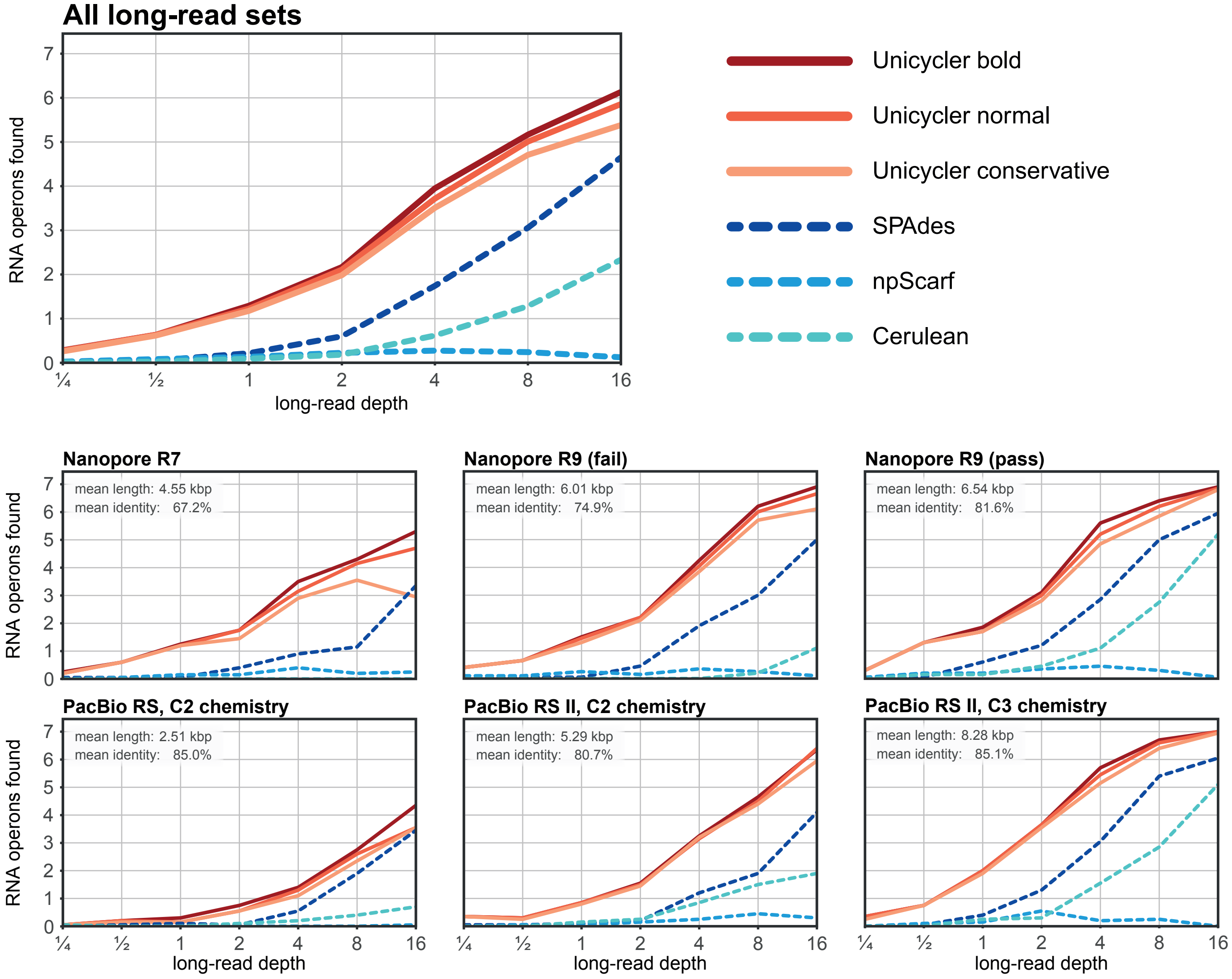
All long-read sets
RNA operons found
long-read depth
Unicycler bold
Unicycler normal
Unicycler conservative
SPAdes
npScarf
Cerulean
Nanopore R7
mean length: 4.55 kbp
mean identity: 67.2%
Nanopore R9 (fail)
mean length: 6.01 kbp
mean identity: 74.9%
Nanopore R9 (pass)
mean length: 6.54 kbp
mean identity: 81.6%
PacBio RS, C2 chemistry
mean length: 2.51 kbp
mean identity: 85.0%
PacBio RS II, C2 chemistry
mean length: 5.29 kbp
mean identity: 80.7%
PacBio RS II, C3 chemistry
mean length: 8.28 kbp
mean identity: 85.1%
