# Supplementary material for: Unicycler: Resolving bacterial genome assemblies from short and long sequencing reads
Source: PLoS Comput Biol. 2017 Jun 8;13(6):e1005595. doi: 10.1371/journal.pcbi.1005595 (PMC5481147; doi:10.1371/journal.pcbi.1005595)
Supplement: S2 Fig — (PDF) [file pcbi.1005595.s002.pdf]

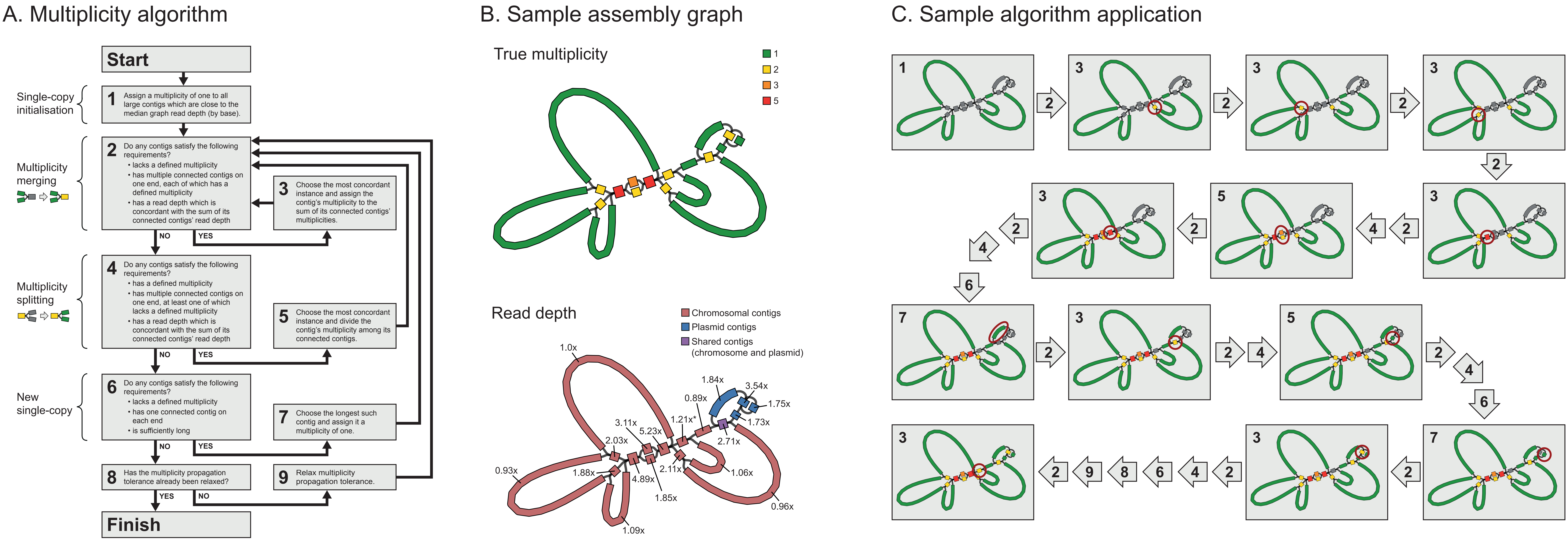

The algorithm begins by assigning single-copy status to large contigs near the median graph read depth (step 1). This covers most large chromosomal contigs. It then propagates that depth through the graph using a greedy algorithm which merges and splits multiplicity where read depth and graph connections are in best agreement (steps 2–5). When no more propagation is possible, it assigns single copy status to the largest candidate single copy contig (steps 6–7) and tries propagation again. This allows single copy status to be assigned to plasmid contigs which are higher than chromosomal depth.

The allowed lengths and read depth tolerances are predefined settings in Unicycler. When no more propagation is possible, these tolerances are relaxed (steps 8–9) so multiplicity can be assigned to contigs with aberrant read depth.

In this simplified example, there are 19 contigs from two replicons (chromosome and plasmid). The read depths are normalised to the graph's median depth (by base). Within a single replicon, read depth is well correlated with the true multiplicity.

The relationship between read depth and multiplicity does not hold between replicons. For example, a single copy contig in the plasmid (1.84x) can have very similar read depth to a repeat contig in the chromosome (1.85x).

* This contig has aberrant read depth which is in poor agreement with its multiplicity.

This diagram follows the sample assembly graph through the algorithm, illustrating each time a contig is assigned multiplicity. The numbers correspond to the steps in the algorithm flow chart. It begins at the top left and ends at the bottom left. Multiplicity first propagates through most chromosomal contigs (first two rows). Step 7 then assigns single copy status to higher read depth contigs, allowing propagation to continue in plasmid contigs.

The contig with aberrant read depth is the last to be assigned. Multiplicity cannot propagate to this contig until the tolerances are relaxed in step 9. By using the information in graph connections, the algorithm correctly assigns this contig a multiplicity of two, despite its unusually low read depth.
