# Supplementary material for: Unicycler: Resolving bacterial genome assemblies from short and long sequencing reads
Source: PLoS Comput Biol. 2017 Jun 8;13(6):e1005595. doi: 10.1371/journal.pcbi.1005595 (PMC5481147; doi:10.1371/journal.pcbi.1005595)
Supplement: S10 Fig — Comparison of assembly graphs made by different assemblers using only short reads for K. pneumoniae INF125. Contigs are coloured based on which replicon they represent. (PDF) [file pcbi.1005595.s010.pdf]

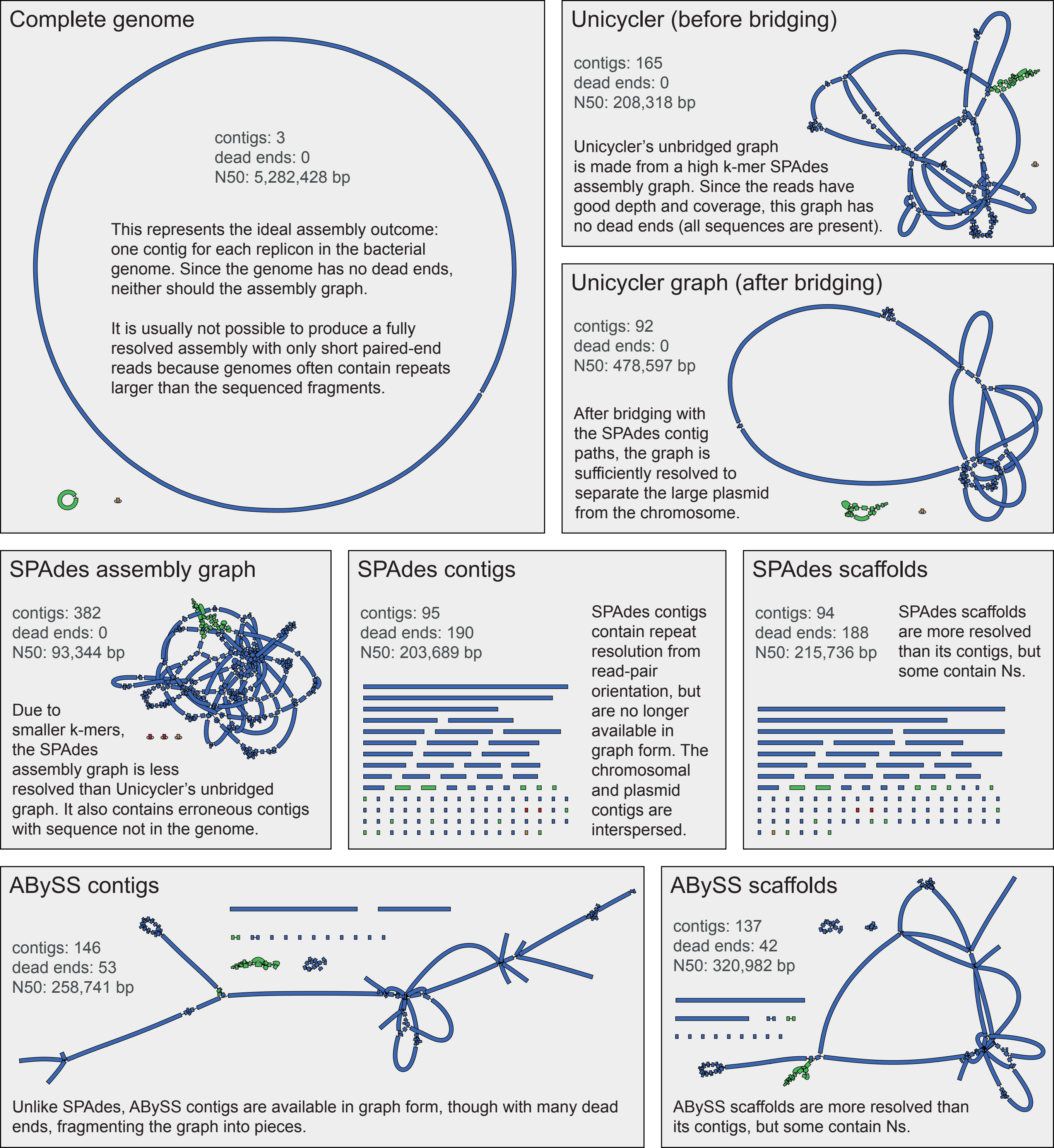
Complete genome
contigs: 3
dead ends: 0
N50: 5,282,428 bp
This represents the ideal assembly outcome: one contig for each replicon in the bacterial genome. Since the genome has no dead ends, neither should the assembly graph.
It is usually not possible to produce a fully resolved assembly with only short paired-end reads because genomes often contain repeats larger than the sequenced fragments.
Unicycler (before bridging)
contigs: 165
dead ends: 0
N50: 208,318 bp
Unicycler's unbridged graph is made from a high k-mer SPAdes assembly graph. Since the reads have good depth and coverage, this graph has no dead ends (all sequences are present).
Unicycler graph (after bridging)
contigs: 92
dead ends: 0
N50: 478,597 bp
After bridging with the SPAdes contig paths, the graph is sufficiently resolved to separate the large plasmid from the chromosome.
SPAdes assembly graph
contigs: 382
dead ends: 0
N50: 93,344 bp
Due to smaller k-mers, the SPAdes assembly graph is less resolved than Unicycler's unbridged graph. It also contains erroneous contigs with sequence not in the genome.
SPAdes contigs
contigs: 95
dead ends: 190
N50: 203,689 bp
SPAdes contigs contain repeat resolution from read-pair orientation, but are no longer available in graph form. The chromosomal and plasmid contigs are interspersed.
SPAdes scaffolds
contigs: 94
dead ends: 188
N50: 215,736 bp
SPAdes scaffolds are more resolved than its contigs, but some contain Ns.
ABySS contigs
contigs: 146
dead ends: 53
N50: 258,741 bp
Unlike SPAdes, ABySS contigs are available in graph form, though with many dead ends, fragmenting the graph into pieces.
ABySS scaffolds
contigs: 137
dead ends: 42
N50: 320,982 bp
ABySS scaffolds are more resolved than its contigs, but some contain Ns.
